# Effects of a Plant-Based Multi-Strain *Limosilactobacillus fermentum* Probiotic on Weight Loss Outcomes in Overweight and Obese Adults: A Preliminary Study

**DOI:** 10.3390/nu18121908

**Published:** 2026-06-12

**Authors:** Sarah Johnson, Broderick L. Dickerson, Jisun Chun, Olivia Haskell, Elena Chavez, Leah Kirkegaard, Kelly Elizabeth Hines, Choongsung Yoo, Joungbo Ko, Dante Xing, Martin Purpura, Ralf Jäger, Ryan J. Sowinski, Drew E. Gonzalez, Christopher J. Rasmussen, Richard B. Kreider

**Affiliations:** 1 Exercise & Sport Nutrition Lab, Department of Kinesiology and Sport Management, Texas A&M University, College Station, TX 77843, USA; sjohnson2216@tamu.edu (S.J.); dickersobl5@tamu.edu (B.L.D.); chunjs3112@tamu.edu (J.C.); oliviajhaskell@tamu.edu (O.H.); ebchavez_10@tamu.edu (E.C.); kirkegaard@tamu.edu (L.K.); khines3@tamu.edu (K.E.H.); choongsungyoo@tamu.edu (C.Y.); joungboko10@tamu.edu (J.K.); dantexing@tamu.edu (D.X.); rjs370@tamu.edu (R.J.S.); dg18@tamu.edu (D.E.G.); crasmussen@tamu.edu (C.J.R.); 2 Department of Kinesiology, School of Natural Sciences, St. Edward’s University, Austin, TX 78704, USA; 3 Emergency Responder Human Performance Laboratory, Exercise and Nutrition Sciences, University at Buffalo, Buffalo, NY 14214, USA; 4 Department of Obstetrics, Gynecology and Reproductive Sciences, University of Maryland School of Medicine, Baltimore, MD 21201, USA; 5 Department of Heart, Blood, and Kidney Research, Cleveland Clinic, Cleveland, OH 44195, USA; 6 Department of Exercise and Sport Science, University of Wisconsin-La Crosse, La Crosse, WI 56401, USA; 7 Increnovo LLC, Whitefish Bay, WI 53217, USA; martin.purpura@increnovo.com (M.P.); ralf.jaeger@increnovo.com (R.J.); 8 Occupational, Performance, and Nutrition Lab, Department of Kinesiology, Sam Houston State University, Huntsville, TX 77340, USA

**Keywords:** diet, body composition, visceral adiposity, bone

## Abstract

Background/Objectives: Multi-strain *Limosilactobacillus fermentum* supplementation has been reported to promote weight loss outcomes in free-living conditions, but limited evidence exists on these probiotic strains added to an energy-restricted diet and walking program in overweight adults. Methods: In a double-blind, placebo-controlled, parallel-arm randomized trial, overweight adults (35.2 ± 13.2 years old, 167.6 ± 8.6 cm, 79.9 ± 11.8 kg, 28.4 ± 2.7 kg/m^2^ body mass index, 36.1 ± 6.6% body fat) completed a 12-week weight loss program that included a 500 kcal/day energy deficit and walking 10 k steps/d. Participants ingested one daily capsule containing a three-strain probiotic blend (*L. fermentum* K7-Lb1, *L. fermentum* K8-Lb1, *L. fermentum* K11-Lb3; 6 billion CFU/day) (PRO) or maltodextrin placebo (PLA). Assessments were performed at baseline, week 6, and week 12 and included body composition, resting energy expenditure, substrate utilization, peak oxygen uptake, dietary intake, step counts, blood biomarkers, quality of life, and side effects. Data were analyzed using multivariate and univariate repeated-measures general linear models (GLM), with mean changes from baseline presented alongside 95% confidence intervals. Results: All participants significantly reduced body weight, fat mass, body fat percentage, and waist circumference. At 12 weeks, PRO reduced fat mass more than PL (−2680.7 ± 1276.7 g; *p* = 0.039). In PRO, android and gynoid fat percentage decreased at 6 weeks (*p* < 0.001; *p* = 0.008) and 12 weeks (*p* = 0.004; *p* < 0.001), respectively. Visceral adipose tissue mass, volume, and area were lower at 6 weeks and trended lower at 12 weeks. In PRO, bone mineral content and bone mineral area decreased at 12 weeks, while bone mineral density paradoxically increased (0.007 ± 0.003 g/cm^2^; *p* = 0.024). Conclusions: During a 12-week weight loss program, supplementation of a multi-strain *L. fermentum* probiotic significantly reduced body fat and central adiposity.

## 1. Introduction

Obesity represents one of the most pressing public health pandemics, contributing to global morbidity, mortality, and economic burden. Global estimates report that obesity prevalence more than doubled between 1980 and 2014, with roughly 15% of adults classified as obese [1]. In the United States, more than 40% of adults are currently considered obese, with projections indicating continued growth [2]. Worldwide, approximately 38% of the global population was classified as overweight or obese in 2020, with trends estimating that over half of the global population may be considered obese by 2035 [3]. Though the United States exhibits the highest prevalence rates of obesity, incidence is markedly increasing across established and developing nations, transforming what was prior considered a problem of affluent societies into a global pandemic [4].

Numerous countermeasures (i.e., exercise, energy-restriction, etc.) against obesity and its associated comorbidities have been researched for years. Recent research indicates that the gut microbiota plays a pivotal role in mediating the relationship between dietary patterns and host metabolic and physiological processes [5,6,7]. Moreover, overweight and obesity have been found to be associated with alterations in both the diversity and composition of the gut microbial community [8,9,10,11,12]. To address these interactions, recent researchers have explored the effects of probiotics supplementation on outcomes associated with obesity and weight loss [13,14,15,16]. Generally, studies show probiotic supplementation to be effective in managing weight loss [13,16,17], ameliorating cardiovascular risk factors [18,19,20,21], blunting inflammation [22,23,24], and correcting dysbiosis associated with obesity [25,26], but results may be species and strain-specific [17].

Kimere, a fermented pearl millet dough common among the Mbeere community of Kenya, contains high concentrations of viable fermentative microorganisms such as different strains of the probiotic *Limosilactobacillus fermentum* (*L. fermentum*) [27,28]. Evidence from Kenya shows children who consumed a locally created, Kimere-based yogurt reduced the urine concentrations of aflatoxin B1, a carcinogenic produced by fungi [29]. Additionally, preclinical models suggest administration of *L. fermentum* 139, 263, 296, and MG4295 exerts antioxidant, anti-inflammatory, and anti-obesogenic effects along with alleviating cardiometabolic risk factors in rats fed high-fat diets [30,31,32]. Although research with *L. fermentum* supplementation is limited in humans, one study does provide evidence that daily supplementation of 5.5 B colony-forming units (CFU) of *L. fermentum* K7-Lb1, K8-Lb1, and K11-Lb3 for 12 weeks significantly improved body composition, visceral adiposity, liver steatosis, and related metabolic parameters in overweight adults [8], thereby supporting their beneficial role in ameliorating inflammation-mediated characteristics of metabolic syndrome associated with excess body weight. Though these findings are interesting, the participants did not undergo a strategic weight loss intervention coupled with probiotic supplementation, which might have elicited additive benefits to the reductions in body weight and visceral fat. Therefore, more research is needed to assess the efficacy of *L. fermentum* supplementation on weight loss-associated outcomes in individuals participating in a weight loss intervention.

This study sought to evaluate the efficacy of a plant-based, multi-strain probiotic supplement (*L. fermentum* K7-Lb1, K8-Lb1, and K11-Lb3; 6 B CFU) as an adjunct to a 12-week weight reduction program consisting of walking-based physical activity (10,000 steps per day) and a 500 kcal/day caloric deficit, in improving weight loss and affecting broader health outcomes among adults with overweight and/or obesity. This research aimed to address changes in body composition, anthropometrics, dietary intake, energy expenditure, and cardiovascular/cardiometabolic risk factors in response to daily probiotic supplementation.

## 2. Materials and Methods

### 2.1. Experimental Design

This study was conducted in a double-blind, placebo-controlled, parallel-arm, randomized manner. The Human Protection Institutional Review Board (IRB2023-0472F) approved this research, which adhered to Declaration of Helsinki ethical standards for human participant research. The trial was also registered through the UK’s Clinical Study Registry (ISRCTN72565988, submitted 03/06/2026, registered 03/10/2026). Nutritional supplementation of a plant-based three-strain probiotic (PRO) blend served as the independent variable. Primary outcome variables consisted of body composition (body mass, fat mass, fat free mass, percent body fat, bone mineral density) and anthropometrics (waist and hip circumferences). Secondary outcomes consisted of maximal aerobic capacity, cell blood counts, comprehensive metabolic panel, physical activity, diet, resting energy expenditure, subjective perceptions of quality of life, and side effects and adverse reactions.

### 2.2. Participants

All participants provided written informed consent prior to study participation. Overweight and obese individuals participated in the study. Inclusion criteria for study participation included (1.) being 18 to 65 years old at the time of consent; (2.) having the ability to comply with the study procedures; (3.) having a willingness to provide voluntary, written, informed consent to participate in the study; (4.) having the ability to complete the study based on the durations of individual visits and scheduling requirements; (5.) being free-living (living in a private home and able to maintain their health and hygiene without assistance); (6.) being in generally good health and willing to participate in a fitness program that includes aerobic walking; and (7.) having a Body Mass Index (BMI) between 25–32 kg/m^2^ and/or body fat > 30%. Exclusion criteria consisted of (1.) women who were pregnant, breastfeeding, or wished to become pregnant during the study (confirmed via a negative pregnancy test); (2.) individuals planned major changes in lifestyle (i.e., diet, dieting, exercise level, travel, etc.) during the study; (3.) Exhibited a recent history (<3 months) of exercise training or weight loss (>5%); (4.) Exhibited an orthopedic limitation that would prevent participation in a general fitness program; (5.) Had uncontrolled heart disease, hypertension, diabetes, thyroid disease, cancer, neurological disease, or untreated psychotic or major depressive disorder in which participation in a general fitness program is contraindicated; (6.) Had taken weight loss dietary supplements or medications in the past 4 weeks preceding study participation; (7.) Had a history of chronic use of oral or injectable corticosteroids; (8.) Showed a history within the previous 12 months of alcohol or substance abuse; (9.) Were a heavy smoker (>1 pack/day within the past 3 months); and (10.) Exhibited a known allergy to any of the ingredients in the supplement product.

**Figure 1:**
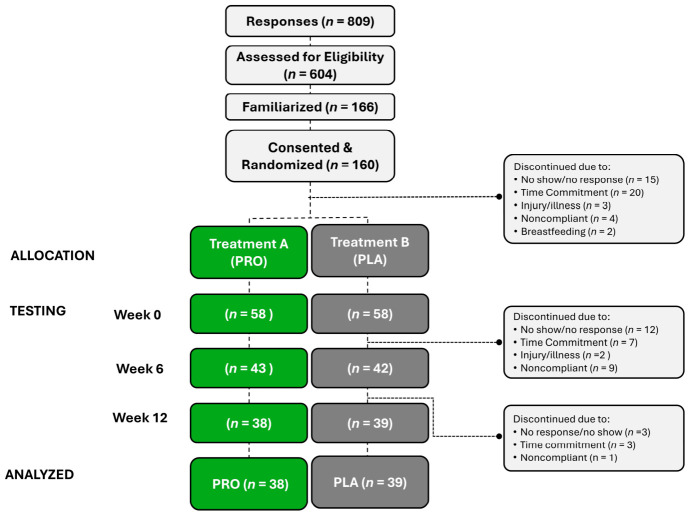
CONSORT Flow Diagram. Post-study unblinding revealed that Treatment A was the probiotic group (PRO) and Treatment B was the placebo (PLA) group.

A Consolidated Standards of Reporting Trials (CONSORT) flow diagram is presented in Figure 1. In total, 809 individuals responded to initial study advertisements, with 604 completing general eligibility assessment to evaluate preliminary study eligibility. Of those, 166 were eligible to be invited to the testing facility for an in-person familiarization to assess final study eligibility. From there, 160 individuals were consented and randomized to begin study procedures. Initially, due to the double-blind nature of the supplement administration, the groups were identified as ‘Treatment A’ and ‘Treatment B’ throughout the duration of data collection and analysis. After analysis was conducted, treatments were then unblinded and revealed treatments A and B were PRO and PLA, respectively (Figure 1). Of the allocated individuals, 15 did not respond to follow-up scheduling consultations, 20 discontinued interest in the study due to time constraints, three either fell ill or sustained injuries outside of the study, four were noncompliant to study procedures, and two were breastfeeding. These drops resulted in 116 individuals being tested at baseline. After baseline, 12 more individuals did not respond to follow-up testing emails or phone calls, seven more were discontinued due to time constraints, two either fell ill or sustained injuries that prevented from continued study participation, and nine more were noncompliant to study procedures and were dropped leaving 85 participants that were tested at week 6. After week 6 testing, three more stopped responding to communications made by the researchers, three dropped due to further time commitment issues, and one was dropped for deviating from study protocols. Resultantly, 77 total participants completed the study, with 38 individuals (male, *n* = 16; female, *n* = 22) finishing in the PRO group and 39 (male, *n* = 11; female, *n* = 28) finishing in PLA.

### 2.3. Study Timeline

**Figure 2:**
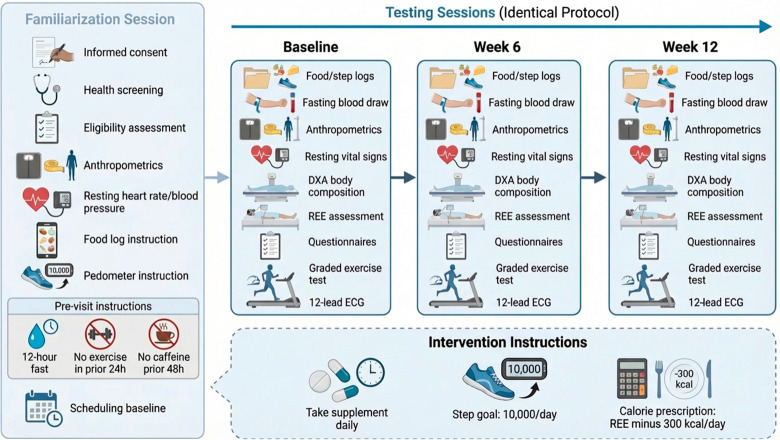
Study Timeline and Procedures. DXA = Dual energy X-ray absorptiometry, ECG = Electrocardiography, REE = Resting Energy Expenditure. Figure created with FigureLabs.ai and ChatGPT 5.2 (OpenAI, San Francisco, CA, USA).

The study timeline and measurements taken during each study visit are shown in Figure 2. Recruitment was conducted through mass e-mail strategies, social media postings, and physical and online flyers distributed through local media. Interested individuals preliminarily underwent phone and/or online screening to assess their general eligibility for the study. Those who met initial screening eligibility were further consulted during an in-person familiarization session, in which they received information about the study protocol, were familiarized with study procedures, and signed voluntary informed consent to participate. Those eligible for study participation after the familiarization session were then randomized into the study and received instructions on how to document their physical activity and dietary intake. Participants were instructed to document their daily walking habits (steps/d) and fluid and food intake 4 days prior and fast for at least 12 h prior to their scheduled baseline testing. After baseline testing, participants were asked to document daily walking habits each day between testing sessions. At baseline, participants brought their 4-day food and beverage logs and walking logs to the testing laboratory, donated a fasting blood sample, completed questionnaires regarding subjective perceptions of quality of life and side effects. Subsequently, participants had resting heart rate (HR) and blood pressure (BP) and waist and hip circumference measurements taken. Participants were then weighed, had body composition and bone density assessed, completed a resting energy expenditure (REE) assessment via indirect calorimetry, and completed a symptom-limited graded, maximal, cardiopulmonary aerobic capacity test. Afterwards, participants were randomly allocated to one of two treatment groups. In a double-blind, randomized, counterbalanced manner, participants were administered either a three-strain probiotic blend (PRO) or maltodextrin placebo (PL). Participants participated in a 12-week dietary and walking intervention that involved reducing daily energy intake by 500 kcals/d based on baseline REE and walking 5 days/week at least 30 min/d, which was monitored via fitness apps and pedometers. Study personnel held weekly consultations with participants to evaluate adherence and repeated all testing procedures at the 6- and 12-week time points of the study intervention.

### 2.4. Dietary Program

Participants were asked to maintain a daily energy intake of 1200, 1300, 1400, 1500, or 1600 kcals based on their baseline REE. The diets given to participants were designed to promote a 300 kcal/d decrease in energy intake from the REE to subtract a 500 kcal/d total energy expenditure with increased physical activity from the walking intervention. The dietary programs administered to the participants were based on American Heart Association guidelines for total daily macronutrient distribution (55% carbohydrate, 15% protein, and 30% fat). At baseline, participants were given a food exchange list that allowed them to manipulate their dietary choices to adhere to their specific daily caloric goals while introducing variety to their foods. In addition to the target daily energy intake goals, participants were provided with examples of meals that were designed to fit their energy intake goals and written weekly dietary plans.

### 2.5. Physical Activity

Participants were instructed to accumulate approximately 10,000 steps/d by participating in a walking program (5 days/week, roughly 30 min/d). Personal phones, pedometers, physical exercise logs, and activity monitors were used by the participants to document the walking activities. Walking logs were collected at each testing session.

### 2.6. Supplementation Protocol

Experimental supplements were administered to participants in a double-blind manner and randomized manner and used an adaptive randomization (minimization) method wherein the first participant was assigned to a group with simple randomization, while the subsequent participants were assigned according to their height, weight, age, sex, and BMI in comparison to existing group assignments, with the goal of balancing the averages of these covariates between the two groups [33]. The experimental treatments were manufactured by MeriCal^®^ (Orange, CA, USA) and consisted of a powdered form of a plant-based, three-strain probiotic blend containing *L. fermentum* K7-Lb1 (DSM 22831), *L. fermentum* K8-Lb1 (DSM 22832), *L. fermentum* K11-Lb3 (DSM 22838) (SlimBiotics^®^ GmbH, Vienna, Austria), providing 2 billion colony-forming units (CFU) per strain [27,34,35]. The strains were originally isolated from Kimere, a traditional fermented pearl millet product consumed in East Africa and are therefore considered plant-derived probiotic strains [27,28,29]. The PLA consisted of a powdered, color, and flavor-matched maltodextrin (Maltrin^®^, Grain Processing Corporation, Muscatine, IA, USA). Each treatment was encapsulated in a white, opaque cellulose capsule shell (ACG Associated Capsules Pvt. Ltd., Madhya Pradesh, India). Supplements were packaged and distributed in identically labeled white bottles to ensure double-blind administration. Contents and purities of the supplements were analyzed by the supplement manufacturers to ensure supplements were unadulterated and safe for human consumption. Participants were instructed to ingest one capsule per day at breakfast with 8–12 ounces of water for 12 weeks. The dosing of the PRO delivered 6 B CFU of the cumulative strains and was determined based on similar dosages being administered in previous in vitro studies [27,34,35].

### 2.7. Participant Demographics

Height and weight were measured with a calibrated digital scale (Health-O-Meter Professional 500KL, Pelstar LLC, Alsip, IL, USA). Following 5 minutes of rest, seated participant hemodynamics were obtained through brachial artery auscultation using a stethoscope and sphygmomanometer according to standard procedures [36]. A soft Gulick tape measure was used to measure waist and hip circumferences using previously established protocols [37].

### 2.8. Diet and Physical Activity

Participants used the MyFitnessPal phone application (MyFitnessPal Inc., Baltimore, MD, USA) [38] to monitor their daily food intake. The 4-day averages of caloric, carbohydrate, protein, and fat intake from the 4-day dietary logs provided by each participant at each testing session were used for subsequent analysis. Daily step counts were monitored using wearable step-tracking devices. Participants were instructed to wear the device during waking hours and to record daily totals. Step data were reviewed at follow-up visits to assess adherence to the prescribed walking program. Average daily step counts were calculated for each assessment period and used to evaluate compliance with the physical activity intervention.

### 2.9. Body Composition

Body composition and bone mineral density (BMD) and content (BMC) were determined using a Hologic Discovery W dual-energy X-ray absorptiometer (Hologic Inc., Waltham, MA, USA) outfitted with APEX version 4.0.2 software (APEX Corporation Software, Pittsburgh, PA, USA) [39,40]. Coefficient of variation (CV) for conducting daily scans in our laboratory has ranged from 0.31–0.45% for BMC and total masses, with a mean intraclass correlation (ICC) of 0.98 [41].

### 2.10. Phlebotomy and Analysis

Certified phlebotomists obtained fasting blood samples at each testing visit using stand phlebotomy techniques [42]. Blood samples were collected in two serum separation tubes (SST) and one ethylenediaminetetraacetic acid (EDTA) (BD Vacutainer, Becton, Dickinson and Company, Franklin Lakes, NJ, USA). Serum separation tubes were allowed to clot at room temperature for 20 min prior to centrifugation at 3000× *g* for 10 min at 4 °C in a Thermo Scientific Heraeus MegaFuge 40R centrifuge (Thermo Scientific North America LLC, West Palm FL, USA). Serum was aliquoted from one SST into microcentrifuge tubes (Eppendorf, Enfield, CT, USA) in the event of future analysis. The remaining tubes were shipped to Clinical Pathology Laboratory (Austin, TX, USA, CLIA #45D0505003, CAP Accreditation #21525-01) for analyses on cell blood count with differential on whole blood and comprehensive metabolic panel and hemoglobin A1C with serum.

### 2.11. Resting Energy Expenditure

Resting metabolic rate was measured using ParvoMedics TrueOne 2400 Resting metabolic measurement system (ParvoMedics Inc., Sandy, UT, USA). Calibration of the system was conducted using a series 5530 3-liter syringe (Hans Rudolph, Kansas City, MO, USA) prior to each testing session. During the REE assessment, participants were placed in a supine position with their knees and hips bent at 90° on a soft cushion. Participants were instructed to stay awake for 20–30 min. After the initial 10 min, the final five consecutive time points that showed less than 5% variance, corresponding to a fractional expired content of carbon dioxide (FeCO_2_) ranging from 1–1.2%, were used to calculate [43,44]. Fatty acid and glucose utilization were estimated by calculating respiratory quotient (i.e., respiratory exchange ratio) [45,46]. A previous study in female athletes documented a CV of 5.3% with an ICC of 0.92 [47] with a manufacturer-reported CV of 2% for healthy populations.

### 2.12. Maximal Aerobic Capacity

The Bruce protocol [48] was employed to assess maximal aerobic capacity, with participants exercising on a motorized treadmill (Trackmaster 425, Trackmaster Treadmills, Newton, KS, USA) until they reached volitional exhaustion. Expired ventilation and oxygen concentration were measured using a metabolic cart (TrueOne 2400, ParvoMedics, Inc., Sandy, UT, USA). The pneumotach was calibrated using a Series 5530 calibration syringe (Hans Rudolph Inc., Kansas City, MO, USA), and the CO_2_ and O_2_ sensors were calibrated with certified medical-grade gases according to the manufacturer’s guidelines. Heart rate and rhythm were monitored via a Cardio-Card version 7.2 electrocardiograph (Nasiff Associates, Brewerton, NY, USA). Perceived exertion and fatigue were recorded using the Borg 6–20 Rating of Perceived Exertion (RPE) scale. Upon reaching volitional fatigue, participants completed a 10 min cool down.

### 2.13. Quality-of-Life Questionnaire

Subjective perceptions of quality of life were assessed using the Short Form Health Survey version 2 (SF36v2). This questionnaire includes broad questions covering multiple domains of physical and psychological well-being, such as mental and emotional health, social functioning, and vitality. Prior research has demonstrated strong test–retest reliability for the SF-36v2, with correlation coefficients ranging from r = 0.81 to 0.85 across all domains [49,50].

### 2.14. Side Effects and Adverse Events Assessment

A self-reported side effects questionnaire was used to determine whether participants experienced any adverse reactions related to their assigned treatments. Participants evaluated both the frequency (F) and severity (S) of any side effects they encountered during the study, such as gastrointestinal (GI) distress, constipation, diarrhea, fatigue, abdominal discomfort, nausea, headache, heartburn, or other adverse events, following previously validated methods [51,52]. In previous assessments conducted in our laboratory, CV for responses to these items ranged from 1.2% to 2.6%, indicating strong reliability [52].

### 2.15. Statistical Analysis

All data analyses were performed using SPSS^®^ Version 31 (IBM Corp., Armonk, NY, USA). Sample size estimation was guided by multiple considerations. First, prior studies from our laboratory examining nutritional interventions in conjunction with weight loss have shown that roughly 20–40 participants per group are typically sufficient in two-group designs to detect statistically significant and practically meaningful effects on weight loss outcomes [51,53,54,55,56,57,58,59]. Second, estimates were informed by reported means, standard deviations, and observed between-group differences from related literature, assuming 80% statistical power, variability of approximately 5–10% relative to the mean, and expected improvements of 5–10% in primary outcomes. The initial goal was to recruit and complete 40–50 participants per group; however, attrition reduced the final analyzable sample to 39 participants per group, which was still adequately powered to observe statistically significant differences between treatments.

Total missing data were reported to be only 2.8% of the cumulative dataset due to various reasons (e.g., missed testing sessions, equipment malfunction, etc.). Therefore, missing numerical values, when present, were imputed using series-adjusted means [60], while missing ordinal or nominal survey responses were replaced using the most frequently used response method [61]. This approach was deemed appropriate given the minimal extent of missing data. The methodological literature suggests that imputation of isolated missing values in longitudinal or repeated-measures designs should account for each participant’s observed response pattern over time and remain plausible for the variable being analyzed. Approaches that incorporate within-subject longitudinal information generally perform better than methods that disregard individual response trajectories [62,63,64]. Accordingly, isolated missing observations were replaced with values intended to reflect the participant’s expected response while minimizing the likelihood of materially influencing statistical inference.

All researchers remained blinded as to the group assignments until all data collection was completed and a determination was made that the study was sufficiently powered with an *n* = 39 per group. For results summarized in tables, a mixed-model General Linear Model (GLM) repeated-measures ANOVA was used to evaluate main effects of time (three time points) and group (two groups), as well as time × group interactions, for all continuous variables. All data were also analyzed with sex added as a factor to explore a potential group by sex-by-time interactions; however, no interactions were found. Therefore, only participant demographic results present sex-based effects at baseline. Assumptions of sphericity were tested with Mauchly’s test, and normality was assessed using kurtosis values. When necessary, inflation of the F statistic was corrected using Wilks’ Lambda for multivariate tests and the Greenhouse–Geisser adjustment for univariate tests [65,66]. Pairwise comparisons were conducted with Fisher’s least-significant difference (LSD) tests for predetermined contrasts as more conservative correction strategies may increase the likelihood for Type II error [65,66]. Type I error was set at *p* < 0.05, with *p*-values between 0.05 and 1.0 interpreted as statistical trends approaching statistical significance. Effect sizes were reported using partial eta squared (η_p_^2^), with thresholds of 0.01 for small, 0.06 for medium, and 0.14 for large effects.

Changes from baseline were evaluated with 95% confidence intervals (CIs) to assess clinical significance, in which they were taken in conjunction with the effect sizes, to signify magnitude of effects per variable in each group [67]. Categorical survey responses (nominal or ordinal) were analyzed using Chi-square tests. Data are reported as means ± standard deviations (SD) or, for clinical significance and pairwise comparisons from baseline, as mean changes from baseline (absolute or percent change), presented with lower (LL) and upper (UL) confidence limits. Cumulatively, these analyses were designed to yield a comprehensive interpretation of the data by amalgamating multivariate and univariate analyses, pairwise comparisons, effect size estimations, and clinical changes from baseline rather than only relying on *p*-values [68,69,70].

## 3. Results

### 3.1. Demographics

Participant demographic data are shown in Table S1. Of 77 participants who completed study protocols, mean age was 35.2 ± 13.2 years, with a mean height of 167.6 ± 8.6 cm and mean body weight of 79.9 ± 11.8 kg. Participants had a mean BMI of 28.4 ± 2.7 kg/m^2^, a mean RHR of 67.5 ± 10.6 BPM, a mean systolic blood pressure (SBP) of 117.8 ± 12.1 mmHg, a mean diastolic blood pressure (DBP) of 76.9 ± 8.5 mmHg, a mean waist circumference of 93.9 ± 11.1 cm, a mean hip circumference of 107.1 ± 6.4 cm, a mean body fat % of 36.1 ± 6.6%, and a peak aerobic capacity of 29.2 ± 7.3 mL/kg/min. No significant differences were found between groups; however, significant differences were found between sexes in height, weight, waist circumference, waist-to-hip ratio, SBP, body fat percentage (BF%), and peak aerobic capacity. Subsequent analysis on primary outcome variables did not reveal sex x group x time effects so only group x time effects results are presented.

### 3.2. Energy and Macronutrient Intake

**Figure 3:**
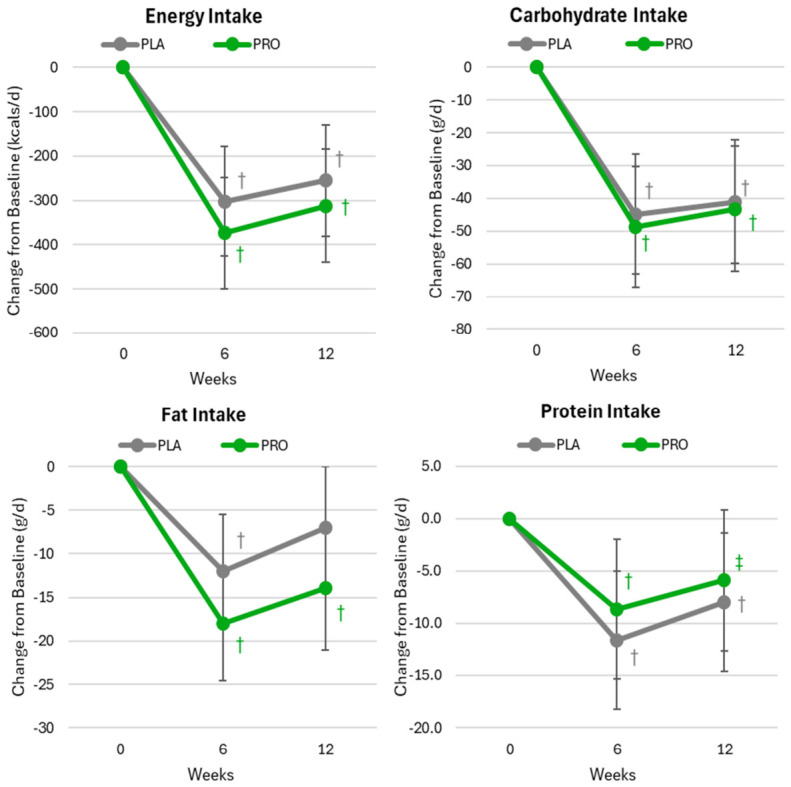
Energy and macronutrient intakes. Data are mean changes from baseline values with 95% confidence intervals. † = *p* ≤ 0.05 diff from baseline values. ‡ = *p* ≥ 0.05–*p* < 0.10 diff from baseline values.

Energy and macronutrient intake is presented in Table S2. Average energy intake was 1398 ± 22 kcals/d, which is commensurate with previous weight loss trials from our lab that induced successful reductions in body mass [51,71,72,73]. Overall, GLM analysis showed significant time (*p* < 0.001, η_p_^2^ = 0.231), but no group x time (*p* = 0.853, η_p_^2^ = 0.014) effects. Univariate analyses showed significant (*p* < 0.001, η_p_^2^ = 0.340) reductions in energy intake from baseline by about −338 kcals/d at week 6 and −284 kcals/d at week 12. No differences in macronutrient intake was observed over the 12 weeks between groups, though univariate analysis showed significant time effects with reductions in carbohydrates (*p* < 0.001, η_p_^2^ = 0.321), dietary fat (*p* < 0.001, η_p_^2^ = 0.233), and protein (*p* < 0.001, η_p_^2^ = 0.114). Figure 3 shows significant decreases from baseline at weeks 6 and 12 in energy and carbohydrates in both groups. Protein intake significantly decreased from baseline at week 6, but this was only a trend at week 12 (*p* = 0.084) in the PRO group. No significant differences were observed between groups in energy or macronutrient intake. These results indicate that both groups adhered to the dietary intervention, and that PRO supplementation did not promote statistically significant changes in self-reported energy or macronutrient intake.

### 3.3. Body Composition Results

**Figure 4:**
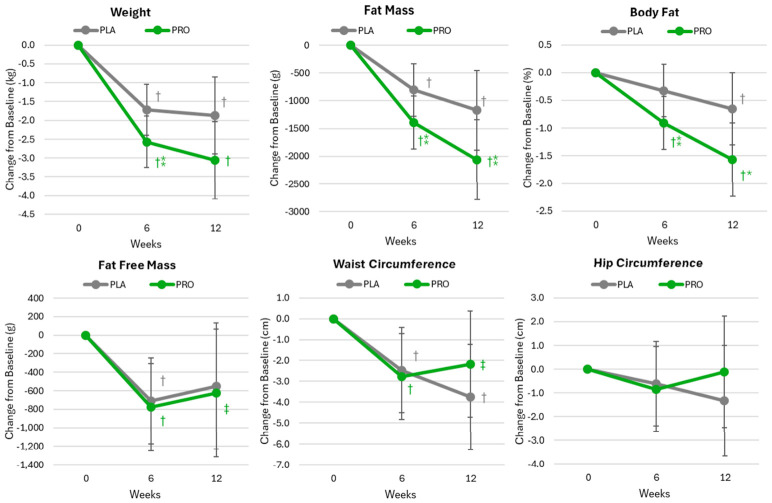
Body Composition and Anthropometrics. Data are mean changes from baseline values with 95% confidence intervals. † = *p* ≤ 0.05 diff from baseline values. ‡ = *p* ≥ 0.05–*p* < 0.10 diff from baseline values. * represents *p* < 0.05 (** = *p* > 0.05 to *p* < 0.10) difference between groups.

Tables S3 and S4 show body composition and anthropometric results, while Figure 4 presents mean changes from baseline. Overall, multivariate analysis showed significant time (*p* < 0.001, η_p_^2^ = 0.248), but no group x time (*p* = 0.224, η_p_^2^ = 0.043) effects for weight, fat mass, BF%, and waist and hip circumference. Multivariate analysis also showed significant time (*p* < 0.001, η_p_^2^ = 0.212), but no group x time (*p* = 0.207, η_p_^2^ = 0.036) effects for total mass, FFM, fat mass, and BF%. Univariate analysis showed significant time effects for weight (*p* < 0.001, η_p_^2^ = 0.384), fat mass (*p* < 0.001, η_p_^2^ = 0.289), BF% (*p* < 0.001, η_p_^2^ = 0.165), waist circumference (*p* < 0.001, η_p_^2^ = 0.102), and FFM (*p* = 0.002, η_p_^2^ = 0.094). Both groups reduced weight, fat mass, BF%, FFM, and waist circumference. Trends were noted for group x time effects for BF% (*p* = 0.085, η_p_^2^ = 0.033). Pairwise comparisons revealed tendencies in differences between groups in weight (−0.852 kg [−0.109, 1.813], *p* = 0.081), fat mass (−583.99 g [−125.47, 87.48], *p* = 0.087), and BF% (−0.58% [−1.25, 0.09], *p* = 0.088) at week 6, with greater reductions shown in the PRO group (Figure 4). This trend continued for fat mass at week 12 in PRO (−891.03 g [−1908.72, 126.65], *p* = 0.085). At week 12, a significant difference was found between groups in BF% (−0.92% [−1.85, 0.00], *p* = 0.05), with PRO showcasing an approximate 1.6% decrease from baseline compared to PLA (−0.65%) (Figure 4).

### 3.4. Visceral Adiposity Tissue Indices

**Figure 5:**
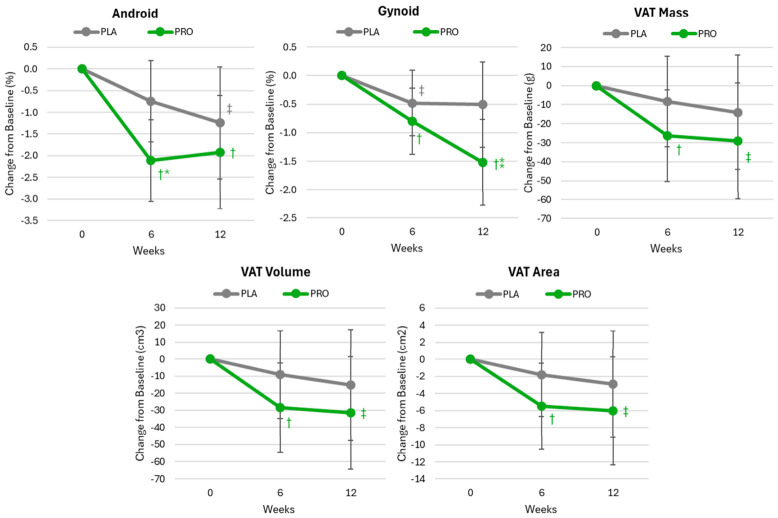
Visceral Adiposity Tissue Measures. Data are mean changes from baseline values with 95% confidence intervals. † = *p* ≤ 0.05 diff from baseline values. ‡ = *p* ≥ 0.05–*p* < 0.10 diff from baseline values. * represents *p* < 0.05 (** = *p* > 0.05 to *p* < 0.10) difference between groups.

Results of visceral adiposity tissue (VAT) measures are presented in Table S5. Overall multivariate analysis showed significant time (*p* < 0.003, η_p_^2^ = 0.088), but no group x time (*p* = 0.405, η_p_^2^ = 0.035) within-subject effects. Univariate analysis showed significant time effects for android (*p* < 0.001, η_p_^2^ = 0.106) and gynoid (*p* < 0.001, η_p_^2^ = 0.111) fat percentage, with both groups experiencing reductions in both metrics at weeks 6 and 12. Although not significant, time effects were demonstrated for VAT (−12.99 g [−24.71, −1.27], *p* = 0.054, η_p_^2^ = 0.040) and VAT volume (−14.02 cm^3^ [−26.69, −1.35], *p* = 0.054, η_p_^2^ = 0.040). However, pairwise comparisons showed the PRO group experienced a significantly greater decrease in android fat percentage at week 6 compared to PLA (−1.37% [−2.69, −0.04], *p* = 0.044) (Figure 5). Delta analyses showed each variable significantly decreased form baseline at week 6 in the PRO group, which was not seen in PLA, although gynoid fat percentage tended to decrease in PLA at week 6 (−0.48% [−1.06, 0.09], *p* = 0.098), and android fat percentage tended to decrease at week 12 (−1.25% [−2.53, 0.04], *p* = 0.057). These significant reductions from baseline in android (−1.92% [−3.23, −0.62], *p* = 0.004) and gynoid fat percentage (−1.52% [−2.27, −0.76], *p* < 0.001) continued for PRO at week 12 with PRO gynoid fat percentage tending to be different than PLA (−1.01% [−2.07, 0.05], *p* = 0.062). VAT mass (−29.07 g [−59.49, 1.35], *p* = 0.061), VAT volume (−31.45 cm^3^ [−64.35, 1.45], *p* = 0.061), and VAT area (−6.02 cm^2^ [−12.33, 0.29], *p* = 0.061) all tended to decrease from baseline at week 12 in PRO (Figure 5).

### 3.5. Bone Measurements

**Figure 6:**
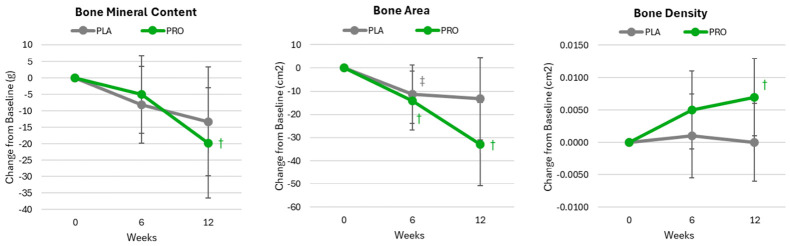
Bone Variables. Data are mean changes from baseline values with 95% confidence intervals. † = *p* ≤ 0.05 diff from baseline values. ‡ = *p* ≥ 0.05–*p* < 0.10 diff from baseline values.

Table S6 presents results on bone mineral content, area, and density. Multivariate analysis showed a significant time (*p* = 0.01, η_p_^2^ = 0.055) effect but no interaction (*p* = 0.558, η_p_^2^ = 0.016) effects. Univariate analysis showed significant time effects for bone mineral content (*p* = 0.011, η_p_^2^ = 0.063) and bone mineral area (*p* = 0.001, η_p_^2^ = 0.101) and a trend for a group effect for bone mineral area (*p* = 0.083, η_p_^2^ = 0.040). Figure 6 shows significant decreases in bone area at weeks 6 (−14.06 cm^2^ [−26.76, −1.36], *p* = 0.031) and 12 (−32.92 cm^2^ [−50.91, −14.92], *p* < 0.001) in PRO. Bone mineral content also significantly decreased at week 12 (−19.79 g [−36.55, −3.04], *p* = 0.021) in PRO, but a significant improvement in bone mineral density was observed (0.007 g/cm^2^, [0.001, 0.013], *p* = 0.024). Additionally, PLA tended to have lower bone area at week 6 compared to baseline (−11.23 cm^2^ [−253.77, 1.31], *p* = 0.078). Although these findings may appear paradoxical, given concurrent decreases in bone mineral content (BMC) and bone mineral area (BMA) alongside an increase in bone mineral density (BMD), the reduction in bone area was proportionally greater than the decrease in BMC, resulting in a net increase in BMD.

### 3.6. Resting Energy Expenditure and Substrate Utilization

Table S7 presents resting energy expenditure and substrate utilization variables. It is typical for REE to decrease concomitant with weight during weight loss; however, no significant time (*p* = 0.441, η_p_^2^ = 0.019) or group x time (*p* = 0.076, η_p_^2^ = 0.784) within-subject effects were found. Avoiding a large reduction in REE is a goal of a weight loss program, which was found in these results. Univariate analysis did not indicate any group, time, or interaction effects in any variables. However, at week 6, carbohydrate oxidation approached a significant decrease (−5.5% [−11.69, 0.53], *p* = 0.073), while fat oxidation tended to increase (5.5% [−0.53, 11.69], *p* = 0.073) from baseline. No significant differences were observed between groups or between time points in REE values.

### 3.7. Physical Activity and Peak Oxygen Uptake

Walking steps/d and peak oxygen uptake results are shown in Table S8. Univariate analysis also revealed a significant time effect (*p* ≤ 0.001, η_p_^2^ = 0.445) for steps taken per day. PRO significantly increased steps/d at weeks 6 (2343.60 steps/d [1656, 3031], *p* ≤ 0.001) and 12 (2413 steps/d [1602, 3224], *p* ≤ 0.001) compared to baseline, with similar results found in the PLA group (2052 steps/d [1382, 2722], *p* ≤ 0.001; 2044 steps/d [1254, 2834], *p* ≤ 0.001, respectively). The increase in steps taken per day in both groups suggest participants successfully adhered to the physical activity requirements of the weight loss program. Multivariate analysis showed a significant time (*p* < 0.01, η_p_^2^ = 0.301), but no group x time (*p* = 0.095, η_p_^2^ = 0.036) effects. Univariate analysis showed a trend for a time effect (*p* = 0.062, η_p_^2^ = 0.038) and a significant group by time effect (*p* = 0.026, η_p_^2^ = 0.050) for absolute peak oxygen uptake, with PRO experiencing significant reductions at weeks 6 (−0.13 L/min [−0.22, −0.04], *p* = 0.006) and 12 (−0.17 L/min [−0.27, −0.07], *p* = 0.001) compared to baseline. Overall, week 12 absolute peak oxygen uptake was significantly lower than baseline (−0.08 L/min [−0.16, −0.02], *p* = 0.014). Univariate analysis also showed a tendency for a group effect in relative peak oxygen uptake, with PLA exhibiting slightly lower values than PRO (0.27 mL/Kg/min [−2.88, 3.43], *p* = 0.083, η_p_^2^ = 0.040), with PLA oxygen uptake tending to be higher at week 6 compared to the baseline (1.52 mL/kg/min [−0.06, 3.1], *p* = 0.060).

### 3.8. Blood Analyses

Table S9 presents complete blood count variables. Multivariate analysis showed no significant time (*p* = 0.112, η_p_^2^ = 0.139 or group x time (*p* = 0.886, η_p_^2^ = 0.078) effects, indicating general clinical safety of the experimental treatments. No interaction effects were noted among all variables. Table S10 presents serum chemistry panel biomarkers. Multivariate analysis showed no significant time (*p* = 0.060, η_p_^2^ = 0.129) or group x time (*p* = 0.090, η_p_^2^ = 0.123) effects. Univariate analysis revealed multiple interaction effects. A group by time effect *(p* = 0.012, η_p_^2^ = 0.059) was revealed for creatinine due to PRO exhibiting elevated baseline values compared to PLA; however, values normalized between groups over time. A tendency for a group by time effect (*p* = 0.078, η_p_^2^ = 0.038) was noted for aspartate aminotransferase with a significant reduction in serum values in the PRO group at week 6 (−1.85 U/L [−3.66, −0.05], *p* = 0.045) and a tendency for a decrease in the PLA group (−1.67 U/L [−3.48, 0.13], *p* = 0.069). Lastly, a tendency for an interaction (*p* = 0.066, η_p_^2^ = 0.038) was identified for estimated glomerular filtration rate (eGFR), with both groups experiencing effects, though at different time points. Results on serum blood lipids are presented in Table S11. Multivariate analysis revealed no significant time (*p* = 0.083, η_p_^2^ = 0.072) or group x time (*p* = 0.338, η_p_^2^ = 0.052) effects. Univariate analyses revealed no group, time, or interaction effects. Table S12 presents effects on serum electrolytes. Multivariate analysis showed no significant time (*p* < 0.895, η_p_^2^ = 0.017) or group × time (*p* = 0.803, η_p_^2^ = 0.021) effects. Univariate analysis also revealed no significant effects. Of note, all blood values at each time point were within clinically normal ranges.

### 3.9. Quality of Life and Perceptions of Side Effects

Table S13 presents quality-of-life responses. Chi-squared analysis revealed significant differences between groups in the two questions. At week 12, perceptions of how health was compared to 1 year ago was significantly different between groups (*p* = 0.022), with more PLA respondents indicating somewhat better health and somewhat worse health compared to PRO at week 12. At baseline and week 6, perceptions of how physical or emotional problems interfered with social activities significantly differed between groups (*p* = 0.012, *p* = 0.040, respectively), with fewer PRO respondents indicating slight or moderate perceptions compared to PLA at both time points. Perceptions tended to differ when asked if health limits moderate physical activities, if participants accomplished less than they would like as a result of any emotional problems, how much of the time does the participant feel full of life, and if they view their health as excellent. Table S14 presents frequency and severity of reported side effects. No significant differences were reported in frequency or severity of responses; however, a tendency toward significance was reported for abdominal discomfort severity at week 12 (*p* = 0.094) and headache severity at week 6 (*p* = 0.054). These results indicate that the supplements investigated were well tolerated.

## 4. Discussion

Increasing physical activity and altering dietary behaviors are often recommended to help promote weight loss, manage symptoms associated with hypokinetic-related disorders, and/or improve overall quality of life in overweight/obese individuals [51,52,57,58,72,74,75,76,77,78,79,80]. Kimere, a spontaneously fermented pearl millet dough common among the Mbeere community of Kenya, East Africa, contain high concentrations of living fermenting microorganisms, with *L. fermentum* dominating the microbiological constituency [27,28]. Previous in vitro and human clinical control trials show administration of isolated microorganisms involved in fermentation promote healthy weight management [8,81,82,83,84,85], improved metabolic health [86,87,88,89], gut barrier integrity [89,90,91], insulin and glucose management [92,93], and promoted microflora balance depleted by antibiotic use [8,81]. However, few data exist on adding strains of *Limosilactobacillus* probiotics to a weight loss program that consists of increasing physical activity levels and adopting a caloric-restricted diet in humans. An additional consideration is the distinction between probiotic supplementation and the consumption of naturally fermented foods. Historically, traditional dietary habits include consuming fermented foods, such as yogurt, kefir, kimchi, sauerkraut, etc., that provide live microorganisms and microbial metabolites. Some evidence suggests that diets rich in minimally processed foods, including fermented foods, is associated with improved health [94,95,96]; however, these dietary patterns differ in Westernized vs. underdeveloped societies [97]. Unlike fermented foods, probiotics deliver specific strains at standardized dosages, allowing for controlled investigation of strain-specific effects. Therefore, the findings noted in the present study should be interpreted as relating specifically to the experimented *L. fermentum* strains and cannot be generalized to fermented foods or other probiotc formulations. Future studies should directly compare probiotc supplementation with fermented food-based interventions to determine whether similar effects on health can be achieved through dietary approaches. This trial assessed if adding a plant-based multi-blend probiotic *(L. fermentum* K7-Lb1 (2 B CFU), *L. fermentum* K8-Lb1 (2 B CFU), and *L. fermentum* K11-Lb3 (2 B CFU)) to a 12-week weight loss program that consisted of walking 10,000 steps per day and undertaking an energy-restricted (−500 kcals/d) diet could promote more favorable weight loss outcomes and health markers in overweight/obese adults. While both groups experienced significant improvements in several weight-loss associated outcomes, participants receiving the probiotic generally demonstrated greater reductions in body fat percentage, fat mass, and certain visceral adiposity measures, although some between-group comparisons only approached statistical significance. These preliminary findings suggest the possibility of modest additive effects of probiotic supplementation during weight loss; however, the results should be interpreted cautiously and confirmed in larger randomized controlled trials. Below, we discuss the primary and secondary outcomes, limitations, and direction of future research.

### 4.1. Primary Outcomes

#### Body Mass, Fat Mass, Visceral Adiposity, and Anthropometrics

Human clinical trials and animal models show supplementation/ingestion of the various *L. Fermentum* strains facilitate weight loss, reduce body fat, improve blood lipid profiles, and blunt inflammation, especially during high-fat diets in rodents [30,82,98,99,100,101,102]. More specifically, administration of *L. fermentum* strains K7-Lb1, K8-Lb1, and K11-Lb3 (5 B CFU each) were recently observed to significantly reduce body mass along with deceasing waist circumference, visceral adipose tissue, and liver steatosis grade compared to a placebo in 180 overweight adults [8]. These changes were in lieu of the participants not modifying lifestyle habits. Proposed mechanisms underlying these effects are based on prior literature and were not directly assessed in the present study. Reportedly, *L. fermentum* induces butyrate production from the gut microbiota, which ultimately stimulates glucagon-like peptide 1 (GLP-I) secretion from the intestinal epithelial enteroendocrine L-cells, thereby stimulating fullness/satiation [8,103,104,105,106]. *L. fermentum* also modulates chaperon Caseinolytic protease B (ClpB), an Enterobacteria-produced protein that mimics α-melanocyte stimulating hormone (α-MSH), an appetite-suppressing peptide and sympathetic nervous system activator [8,107,108]. Collectively, these pathways may contribute to regulation of energy intake, metabolic processes, and fat distribution, potentially explaining the reductions in adiposity observed in the present study. Though these results are promising, Laue et al. [8] is the only study to date in humans testing *L. fermentum* strains K7-Lb1, K8-Lb1, and K11-Lb3 against obesity-related outcomes. Therefore, more clinical trials are warranted on the effects of *L. fermentum* K7-Lb1, K8-Lb1, and K11-Lb3 on weight loss outcomes in overweight/obese adults who increase physical activity and adopt an energy-restricted diet.

In the current study, we examined body composition changes via DEXA after *L. fermentum* K7-Lb1, K8-Lb1, and K11-Lb3 supplementation combined with daily walking and dietary modification. We noted significantly reduced body weight, fat mass, and body fat percentage, fat free mass, and waist circumferences in both groups across 12 weeks. Similar to the findings of Laue and workers [8], body fat percentage was significantly lower in PRO than in PLA at 12 weeks with fat mass and body weight tending to be lower at 12 weeks in PRO. Weight tended to be different in the PRO group from PLA at 6 weeks, but no difference between groups were noted at week 12. Contrarily, fat-free mass did significantly decrease at 6 weeks in both groups and tended to be lower in PRO at 12 weeks. This could be explained by the lack of resistance training in the weight loss program, something that has been reported in previous similar experiments utilizing only aerobic training as the exercise modality [109,110]. Concomitantly, both groups significantly reduced calorie intake over the course of the intervention with a significant reduction in protein intake at 6 weeks and a tendency to be lower at 12 weeks in the PRO group. The omission of resistance training and the significant decreased protein intake over the course of the study could both explain the significant reductions in fat-free mass. Nonetheless, our findings of decreased fat mass, body fat percentage, and body weight while supplementing with a multi-strain probiotic do align with previous reports [8,82,83,84,85].

Additionally, we reported significant reductions in all indices of VAT. Android fat, gynoid fat; VAT mass, volume, and area decreased significantly in PRO compared to baseline, while PLA android fat tended to decrease by week 12. At 6 weeks, PRO exhibited a greater reduction in android fat compared to PLA. These findings align with previous reports indicating decreased visceral fat in response to chronic *L. fermentum* supplementation [8,83]. Coinciding with changes in visceral adiposity were reductions in waist circumference; however, there was no difference between groups at 6 and 12 weeks, with both groups experiencing a significant decrease by week 6. PLA waist circumference continued to decrease by week 12, which was significantly lower than baseline, although PRO waist circumference at week 12 tended to be different from baseline. These findings suggest that supplementation with *L. fermentum* may contribute to modest favorable changes in fat mass and anthropometric measures during a structured weight loss program; however, because several between-group differences only approached statistical significance, these observations should be interpreted with caution.

### 4.2. Secondary Outcomes

#### 4.2.1. Bone Mineral Content, Area, and Density

Weight loss can typically be associated with decrements in bone-related outcomes when no resistance training or appropriate nutrition habits are implemented [111,112,113,114]. In the present study, BMC and bone mineral area significantly decreased from baseline at week 12, though BMD significantly increased in the PRO group. Results from Han et al. [115] mirror these results in which postmenopausal women were supplemented with *L. fermentum* SRK414 (4.0 × 10^9^ CFU) for 6 months and experienced increased BMD of the femoral neck. However, Jafar Nejad and researchers [116] reported no increases in BMD in postmenopausal women supplementing with a multispecies probiotic blend for 6 months. Our data differ from previous trials due to the confounding decreases in BMA and BMC yet increased BMD. Other preclinical models [117,118,119] and systematic reviews [120] do show either bone protective or anti-osteoporotic effects of *Limosilactobacillus* probiotics. The results from our study suggest increases in BMD for individuals supplementing with a multi-strain *Limosilactobacillus* probiotic during a weight loss program; however, these results should be interpreted with caution due to the disproportionate decreases in BMC and BMA. Areal BMD measurements are 2D measurements and are strongly influenced by bone size. Smaller BMA can artifactually elevate BMD values despite reductions in bone mass/content [121,122]. Due to the dearth of evidence with these data, more research is needed to further determine the effects of multi-strain *Limosilactobacillus* probiotics on bone health.

#### 4.2.2. Resting Energy Expenditure and Substrate Utilization

Resting energy expenditure commonly decreases during weight loss programs due to losses in total body mass [123,124,125], contributing to adaptive thermogenesis and difficulty maintaining weight loss. Therefore, maintaining energy expenditure during weight loss interventions is critical in optimizing and sustaining loss of body mass. In the present trial, REE did not significantly change over time in either group despite reductions in total body mass, FFM, and fat mass. Although our study showed no differences in REE between treatments, *L. fermentum* has shown to potentiate brown adipose tissue-mediated energy expenditure in mice, thereby promoting thermogenesis and increased energy expenditure [126]. Although our results do not indicate enhanced REE with *L. fermentum* supplementation, more research is necessary to assess if probiotic supplementation can alter energy expenditure during training or during weight loss programs.

Contrarily, carbohydrate oxidation tended to decrease while fat oxidation increased by week 6 in both groups, though no differences between groups were apparent. These data may indicate early shifts in substrate utilization during weight loss, which has been shown from our lab before [51]. *L. fermentum* LM1016 and CQPC04 has previously been reported to enhance fat oxidation by shifting lipid metabolism toward greater mobilization and β-oxidation, including downregulation of peroxisome proliferator-activated receptor gamma (PPAR-γ), upregulation of carnitine palmitoyl transferase (CPT-I) and cholesterol 7α-hydroxylase (CYP7A1), and increased activity of lipid-regulating transcription factors such as PPAR-α [126,127]. These mechanisms might help explain the shift in substrate metabolism in the early, adaptive period of the weight loss program. However, more research is needed to explore whether PRO supplementation during an exercise and weight loss intervention affects substrate oxidation.

#### 4.2.3. Peak Aerobic Capacity and Functionality

Both groups significantly increased steps/d from baseline at weeks 6 and 12, indicating adherence to the walking program. However, from baseline, absolute peak oxygen uptake decreased in PRO at week 6 (−0.128 L/min, *p* = 0.006) and week 12 (−0.174 L/min, *p* = 0.001), while no changes were observed in PLA. Relative peak oxygen uptake showed a tendency in the PLA group to increase at week 6 and showed an overall trend for a group effect. These findings may reflect the intervention only stressed accumulating roughly 10,000 steps/d, with no emphasis placed on an aerobic exercise component that, in theory, would have elicited a stimulus that could increase aerobic capacity. Some research does suggest an overall benefit for probiotic supplementation on aerobic capacity, specifically in athletes [128,129], but there is a scarcity of evidence on *L. fermentum* strains on eliciting benefits for cardiovascular fitness. Additionally, losses in body mass in PRO could help explain the decrements reported in absolute peak oxygen uptake as relative oxygen uptake did not change over time. This highlights the importance of incorporating aerobic exercise during weight loss to improve cardiovascular fitness. Cumulatively, these data indicate the walking program increased daily activity but did not effectively improve aerobic capacity over 12 weeks in overweight, young-to-middle aged adults; however, more research is needed.

#### 4.2.4. Clinical Safety Biomarkers

Complete blood count variables did not show significant changes over time in PRO, supporting tolerability and safety of supplementation. A small number of time effects were observed for certain white blood cells in PLA; however, there were no differences between groups across variables. All blood counts were also within normal ranges in the PRO group, further supporting safety and tolerability of *L. fermentum* supplementation.

Comprehensive metabolic panel results indicated several significant group and time effects. Glucose was significantly lower in PRO than PLA at all time points, though no differences from baseline were found. In this regard, some evidence does suggest modest improvements in glucose regulation with probiotic supplementation, but effects vary by strain and population [130]. By week 12, creatinine significantly decreased from baseline in PRO with no changes reported for PLA. Aspartate aminotransferase significantly decreased by week 6 in PRO but then tended to be higher than baseline values at week 12. No changes were reported for electrolytes or for blood lipids. All values were within normal reported ranges. Collectively, these findings support that a multi-strain *L. fermentum* supplement is well tolerated and did not adversely affect clinical safety biomarkers during a 12-week weight loss intervention.

#### 4.2.5. Quality of Life and Side Effects

Our results indicate more participants in the PRO group reported perceiving their health as “much better than 1 year ago” at week 12 compared to PLA. In addition, more respondents in PRO reported ‘Not at all’ or ‘Sightly’ when asked ‘to what extent has physical or emotional problems interfered with social activities’ at baseline, though these numbers seemed to change by week 6 compared to PLA. No other significant differences in quality of life perceptions were noted between groups. Side Effect frequency and severity did not differ between groups. No significant changes in onset or manifestation of side effects were noted in PRO, thereby indicating supplementation with a multi-strain *L. fermentum* probiotic appears to be well tolerated. However, additional research evaluating the long-term influence of PRO supplementation on markers of health is warranted.

### 4.3. Limitations and Future Directions

Several limitations can be considered for the present study. First, dietary intake was self-reported and, although dietary adherence appeared strong, underreporting is possible among participants. Second, the intervention did not include structured resistance or aerobic exercise. Coupled with reduced protein intake over time, the lack of resistance and aerobic training may have sacrificed lean body tissue, thereby limiting FFM maintenance and potentially influenced functional and aerobic exercise-related outcomes. Third, mechanistic assays were not performed (e.g., microbiome sequencing, short chain fatty acids, appetite hormones, anti- and pro-inflammatory cytokines), thus limiting insight into potential biological pathways. Fourth, although typical for exercise and diet interventions, the trial experienced high attrition rate due to participant compliance in the weight loss program, time commitment, or other issues. However, attrition between groups was similar. Another limitation is the relatively short duration of the intervention. Although 12 weeks are sufficient to detect early changes in body composition during a regimented weight loss program, it does not permit evaluation of the long-term sustainability of the observed effects. Future studies should investigate whether any benefits associated with *L. fermentum* supplementation during weight loss persists beyond the study period utilized in the present study. This trial also did not include a comparator group that consumed whole, fermented foods. Inclusion of this group would have provided information regarding whether supplementation with specific probiotic strains offers advantages beyond solely increasing the daily consumption of naturally fermented foods. Future trials comparing probiotic supplements with fermented foods may help clarify the efficacy of these dietary approaches. Last, several outcomes only approached statistical significance (*p* > 0.05 to *p <* 0.10), indicating that larger samples may be needed to detect modest between-group effects more consistently and that caution should be exercised when interpreting those findings.

## 5. Conclusions

In overweight and obese adults participating in a 12-week energy-restricted diet and walking intervention, supplementation with a plant-based, three strain *L. fermentum* probiotic blend (6 B CFU/d) was well tolerated, with no clinically significant changes in blood biomarkers. Probiotic supplementation was associated with numerically greater reductions in fat mass, BF%, and measures of central adiposity than placebo, although many between-group differences did not reach statistical significance. Collectively, these findings suggest that targeted *L. fermeneum* supplementation may provide modest additive benefits during lifestyle-based weight loss interventions. However, additional trials are warranted to confirm these effects.

## Data Availability

Data and statistical analyses are available for non-commercial scientific inquiry and/or educational use if request and use do not violate IRB restrictions and/or research agreement terms.

## Supplementary Materials

The following supporting information can be downloaded at: https://www.mdpi.com/article/10.3390/nu18121908/s1, Table S1: Demographic Data; Table S2: Dietary Analysis Results; Table S3: Primary Outcomes—Weight and Anthropometrics; Table S4: DEXA Body Composition Results; Table S5: Visceral Adipose Tissue Analysis; Table S6: Bone Related Variables; Table S7: Resting Energy Expenditure and Substrate Utilization; Table S8: Oxygen Uptake and Steps per Day; Table S9: Complete Blood Count; Table S10: Serum Chemistry Markers; Table S11: Serum Lipid Results; Table S12: Serum Electrolytes; Table S13: Quality of Life Ratings; Table S14a: Frequency of Side Effects; Table S14b: Severity of Side Effects.

## Abbreviations

The following abbreviations are used in this manuscript:

A1C	Hemoglobin A1C
ANOVA	Analysis of variance
AST	Aspartate aminotransferase
α-MSH	Alpha-melanocyte stimulating hormone
BD	Becton, Dickinson and Company
BF%	Body fat percentage
BMI	Body Mass Index
BMC	Bone mineral content
BMD	Bone mineral density
BP	Blood pressure
BPM	Beats per minute
CFU	Colony-forming units
CIs	Confidence intervals
ClpB	Caseinolytic protease B
cm	Centimeters
CONSORT	Consolidated Standards of Reporting Trials
CO_2_	Carbon dioxide
CPT-I	Carnitine palmitoyl transferase I
CPXT	Cardiopulmonary exercise test
CV	Coefficient of variation
CYP7A1	Cholesterol 7α-hydroxylase
DBP	Diastolic blood pressure
DEXA	Dual-energy X-ray absorptiometry
EDTA	Ethylenediaminetetraacetic acid
eGFR	Estimated glomerular filtration rate
FeCO_2_	Fractional expired content of carbon dioxide
FFM	Fat-free mass
GI	Gastrointestinal
GLM	General Linear Model
GLP-I	Glucagon-like peptide 1
HR	Heart rate
ICC	Intraclass correlation
IRB	Institutional Review Board
kcals/d	Kilocalories per day
kg	Kilograms
kg/m^2^	Kilograms per meter squared
L.	Limosilactobacillus
LL	Lower limit (confidence limit)
LSD	Least significant difference
PRO	Probiotic (treatment group; also labeled “PRO, Probiotic”)
PPAR-α	Peroxisome proliferator-activated receptor alpha
PPAR-γ	Peroxisome proliferator-activated receptor gamma
PLA	Placebo
RBP	Resting blood pressure
REE	Resting energy expenditure
RER	Respiratory exchange ratio
RHR	Resting heart rate
RPE	Rating of Perceived Exertion
SBP	Systolic blood pressure
SD	Standard deviation
SF36v2/SF-36v2	Short Form Health Survey version 2
SST	Serum separation tube(s)
steps/d	Steps per day
UL	Upper limit (confidence limit)
VAT	Visceral adiposity tissue
