# Supplementary material for: Effects of a Plant-Based Multi-Strain Limosilactobacillus fermentum Probiotic on Weight Loss Outcomes in Overweight and Obese Adults: A Preliminary Study
Source: Nutrients. 2026 Jun 12;18(12):1908. doi: 10.3390/nu18121908 (PMC13304489; doi:10.3390/nu18121908)
Supplement: Supplementary file 1 [file nutrients-18-01908-s001.zip › nutrients-4346259-supplementary.pdf]

Table S1. Demographic data

Variable	Treatment	Female Mean	Female SD	Female *n*	Male Mean	Male SD	Male *n*	Total Mean	Total SD	Total *n*	Effect	p-Value
Age	PLA	34.0	10.7	28	40.5	16.8	11	35.8	12.8	39	G	0.356
(yrs)	PRO	36.2	13.9	22	32.4	13.7	16	34.6	13.7	38	S	0.667
	Total	34.9	12.2	50	35.7	15.3	27	35.2	13.2	77	G x S	0.110
Height	PLA	164.2	8.1	28	175.5	8.1	11	167.4	9.5	39	G	0.423
(cm)	PRO	163.5	4.7	22	173.5	7.3	16	167.7	7.7	38	S	0.000
	Total	163.9	6.8	50	174.3	7.5	27	167.6	8.6	77	G x S	0.709
Weight	PLA	76.8	12.3	28	89.7	12.5	11	80.4	13.5	39	G	0.258
(kg)	PRO	75.0	7.3	22	85.6	9.6	16	79.5	9.8	38	S	0.000
	Total	76.0	10.3	50	87.3	10.8	27	79.9	11.8	77	G x S	0.295
Body Mass Index	PLA	28.4	3.5	28	29.0	3.0	11	28.6	3.3	39	G	0.464
(kg/m^2)	PRO	28.0	2.1	22	28.4	1.9	16	28.2	2.0	38	S	0.488
	Total	28.2	2.0	38	28.6	2.4	27	28.4	2.7	77	G x S	0.652
Waist	PLA	91.7	9.7	28	103.7	11.0	11	95.1	11.4	39	G	0.076
(cm)	PRO	89.1	10.1	22	97.5	10.2	16	92.6	10.8	38	S	0.000
	Total	90.5	9.9	50	100.0	10.8	27	93.9	11.1	77	G x S	0.864
Hip	PLA	108.0	7.7	28	108.4	6.4	11	108.1	7.3	39	G	0.101
(cm)	PRO	108.3	4.6	22	103.2	4.8	16	106.1	5.3	38	S	0.126
	Total	108.1	6.5	50	105.3	6.0	27	107.1	6.4	77	G x S	0.468
Waist:Hip Ratio	PLA	0.85	0.08	28	0.96	0.10	11	0.88	0.10	39	G	0.401
	PRO	0.82	0.08	22	0.95	0.11	16	0.87	0.11	38	S	0.000
	Total	0.84	0.08	50	0.95	0.10	27	0.88	0.11	77	G x S	0.688
Resting Heart Rate	PLA	67.6	12.8	28	66.7	8.1	11	67.4	11.6	39	G	0.790
(beats/min)	PRO	67.0	10.0	22	68.7	9.6	16	67.7	9.7	38	S	0.885
	Total	67.4	11.5	50	67.9	8.9	27	67.5	10.6	77	G x S	0.632
Systolic Blood Pressure Rate	PLA	114.7	12.6	28	122.4	13.2	11	116.9	13.1	39	G	0.981
(mmHg)	PRO	114.0	10.8	22	122.9	12.1	16	117.8	12.1	38	S	0.006
	Total	114.4	11.8	50	122.7	12.3	27	117.8	12.1	38	G x S	0.827
Diastolic Blood Pressure Rate	PLA	75.8	7.9	28	78.2	8.6	11	76.4	8.0	39	G	0.738
(mmHg)	PRO	75.7	8.2	22	79.6	9.7	16	77.3	8.9	38	S	0.124
	Total	75.7	7.9	50	79.0	9.1	27	76.9	8.5	77	G x S	0.713
Body Fat	PLA	39.5	5.2	28	30.9	4.5	11	37.1	6.3	39	G	0.435
(%)	PRO	39.8	3.9	22	28.9	4.2	16	35.2	6.8	38	S	0.000
	Total	39.6	4.6	50	29.7	4.4	27	36.1	6.6	77	G x S	0.295
Peak Aerobic Capacity	PLA	27.1	7.4	28	31.4	5.5	11	28.4	7.1	39	G	0.400
(ml/kg/min)	PRO	27.0	4.0	22	34.4	8.9	16	30.1	7.4	38	S	0.001
	Total	27.1	6.1	50	33.2	7.7	27	29.2	7.3	77	G x S	0.346

Data are expressed as means, standard deviations for the placebo (PLA) and plant probiotic (PRO) groups. SD = ± standard deviation, G = group, S = sex, G x S = group x sex interaction. Multivariate analysis revealed no overall G (p = 0.791) or G x S (p = 0.688) differences with a significant S difference (p = <0.001).

Table S2. Dietary analysis results.

Variable	Group	N	Weeks 0	Weeks 6		Weeks 12		Mean (SEM)	Effect	p-Level	η_p^2
Energy Intake	PLA	*39*	1587 ± 382	1284 ± 174	†	1332 ± 201	†	1401 ± 31	G	0.873	0.000
(kcals/d)	PRO	*38*	1623 ± 380	1249 ± 230	†	1310 ± 269	†	1394 ± 31	T	0.000	0.340
	Total	*77*	1605 ± 379	1267 ± 203	†	1321 ± 236	†	1398 ± 22	G x T	0.647	0.006
Carbohydrate	PLA	*39*	185.1 ± 62.1	140.2 ± 29.8	†	144.0 ± 30.9	†	156.5 ± 5.0	G	0.933	0.000
(g/d)	PRO	*38*	187.7 ± 57.9	138.9 ± 41.1	†	144.5 ± 30.1	†	157.1 ± 5.1	T	0.000	0.321
	Total	*77*	186.4 ± 59.7	139.6 ± 35.6	†	144.3 ± 30.3	†	156.8 ± 3.6	G x T	0.940	0.001
Fat	PLA	*39*	62.8 ± 19.5	50.8 ± 12.3	†	55.8 ± 12.9	†	56.5 ± 1.8	G	0.577	0.004
(g/d)	PRO	*38*	65.6 ± 21.3	47.6 ± 13.7	†	51.7 ± 16.4	†	55.0 ± 1.9	T	0.000	0.233
	Total	*77*	64.2 ± 20.3	49.2 ± 13.0	†	53.8 ± 14.8	†	55.7 ± 1.3	G x T	0.265	0.018
Protein	PLA	*39*	71.6 ± 21.2	60.0 ± 15.7	†	63.7 ± 16.5	†	65.1 ± 2.4	G	0.862	0.000
(g/d)	PRO	*38*	70.5 ± 19.7	61.9 ± 19.0	†	64.7 ± 22.1	‡	65.7 ± 2.4	T	0.000	0.114
	Total	*77*	71.1 ± 20.4	60.9 ± 17.3	†	64.2 ± 19.3	†	65.4 ± 1.7	G x T	0.814	0.003

Data are expressed as means, standard deviations for the placebo (PLA) and plant probiotic (PRO) groups. Data were analyzed using a multivariate and univariate General Linear Model with repeated measures. P-levels, with partial ETA squared (η_p^2), are listed for between-subject group (G) and univariate within-subject (Greenhouse-Geisser) time (T), and group x time (G x T) effects. Multivariate Wilk's Lambda showed significant time ($p < 0.001$, $\eta_p^2 = 0.231$), but no group x time ($p = 0.853$, $\eta_p^2 = 0.014$) within-subject effects. Pairwise comparisons, with LSD confidence interval adjustment, for simple main effects are indicated by the following superscripts: difference from baseline value, † = p<0.05 (‡ = p>0.05 to p<0.10); and between groups, * = p<0.05 (⁑ = p>0.05 to p<0.10). η_p^2 = effect size where values of 0.01 - 0.05 = small, 0.06 - 0.13 = medium, and >0.14 = large.

Table S3. Primary outcomes - Weight and Anthropometrics

Variable	Group	N	Weeks 0	Weeks 6		Weeks 12		Mean (SEM)		Effect	p-Level	η_p^2
Weight	PLA	*39*	80.41 ± 13.52	78.69 ± 12.84	†	78.54 ± 12.81	†	79.21 ± 1.86		G	0.083	0.040
(kg)	PRO	*38*	79.48 ± 9.77	76.91 ± 9.89	†	76.41 ± 10.46	†	77.60 ± 1.88		T	0.000	0.384
	Total	*77*	79.95 ± 11.75	77.81 ± 11.44	†	77.49 ± 11.68	†	78.40 ± 1.32		G x T	0.111	0.032
Fat Mass	PLA	*39*	28,155 ± 6,444	27,350 ± 6,407	†	26,986 ± 5,952	†	27,497 ± 912		G	0.114	0.033
(g)	PRO	*38*	26,366 ± 5,177	24,976 ± 5,383	†⁑	24,305 ± 5,216	†*	25,216 ± 924	*	T	0.000	0.289
	Total	*77*	27,272 ± 5,884	26,178 ± 6,005	†	25,663 ± 5,725	†	26,356 ± 649		G x T	0.115	0.030
Body Fat	PLA	*39*	37.08 ± 6.34	36.75 ± 6.60		36.43 ± 6.32	†	36.75 ± 1.05		G	0.114	0.033
(%)	PRO	*38*	35.19 ± 6.76	34.29 ± 6.91	†	33.62 ± 6.73	†⁑	34.37 ± 1.06		T	0.000	0.165
	Total	*77*	36.15 ± 6.58	35.54 ± 6.82	†	35.04 ± 6.63	†	35.56 ± 0.74		G x T	0.085	0.033
Waist	PLA	*39*	95.06 ± 11.37	92.59 ± 10.61	†	91.32 ± 11.69	†	92.99 ± 1.65		G	0.391	0.010
(cm)	PRO	*38*	92.62 ± 10.82	89.84 ± 10.48	†	90.44 ± 11.31	‡	90.96 ± 1.67		T	0.000	0.102
	Total	*77*	93.85 ± 11.10	91.23 ± 10.57	†	90.89 ± 11.44	†	91.98 ±		G x T	0.434	0.011
Hip	PLA	*39*	108.1 ± 7.3	107.5 ± 7.5		106.8 ± 7.0		107.5 ± 0.9		G	0.184	0.023
(cm)	PRO	*38*	106.1 ± 5.3	105.3 ± 4.6		106.0 ± 7.4		105.8 ± 0.9		T	0.518	0.009
	Total	*77*	107.1 ± 6.4	106.4 ± 6.3		106.4 ± 7.2		106.6 ± 0.6		G x T	0.562	0.007

Data are expressed as means, standard deviations for the placebo (PLA) and plant probiotic (PRO) groups. Data were analyzed using a multivariate and univariate General Linear Model with repeated measures. P-levels, with partial ETA squared (η_p^2), are listed for between-subject group (G) and univariate within-subject (Greenhouse-Geisser) time (T), and group x time (G x T) effects. Multivariate Wilk's Lambda showed significant time ($p < 0.001$, $\eta_p^2 = 0.248$), but no group x time ($p = 0.224$, $\eta_p^2 = 0.043$) within-subject effects. Pairwise comparisons, with LSD confidence interval adjustment, for simple main effects are indicated by the following superscripts: difference from baseline value, † = p<0.05 (‡ = p>0.05 to p<0.10); and between groups, * = p<0.05 (⁑ = p>0.05 to p<0.10). η_p^2 = effect size where values of 0.01 - 0.05 = small, 0.06 - 0.13 = medium, and >0.14 = large.

Table S4. DEXA body composition results.

Variable	Group	N	Weeks 0	Weeks 6		Weeks 12		Mean (SEM)		Effect	p-Level	η_p^2
Total Mass	PLA	*39*	76541 ± 13492	74770 ± 12574	†	74567 ± 12629	†	75293 ± 1820		G	0.574	0.004
(kg)	PRO	*38*	75449 ± 9485	73280 ± 9728	†	72762 ± 10055	†	73830 ± 1844		T	0.000	0.330
	Total	*77*	76002 ± 11624	74035 ± 11211	†	73676 ± 11393	†	74561 ± 1295		G x T	0.436	0.010
Fat Mass	PLA	*39*	28155 ± 6444	27350 ± 6407	†	26986 ± 5952	†	27497 ± 912		G	0.083	0.040
(g)	PRO	*38*	26366 ± 5177	24976 ± 5383	†⁑	24305 ± 5216	†*	25216 ± 924	⁑	T	0.000	0.289
	Total	*77*	27272 ± 5884	26178 ± 6005	†	25663 ± 5725	†	26356 ± 649		G x T	0.114	0.030
Fat Free Mass	PLA	*39*	48129 ± 10379	47420 ± 10152		47581 ± 10351	†	47710 ± 1565		G	0.686	0.002
(g)	PRO	*38*	49081 ± 9340	48304 ± 9243	†⁑	48457 ± 9371	‡*	48614 ± 1585		T	0.002	0.094
	Total	*77*	48599 ± 9827	47856 ± 9660	†	48013 ± 9824	†	48162 ± 1114		G x T	0.958	0.000
Body Fat	PLA	*39*	37.08 ± 6.34	36.75 ± 6.60		36.43 ± 6.32	†	36.75 ± 1.05		G	0.114	0.033
(%)	PRO	*38*	35.19 ± 6.76	34.29 ± 6.91	†	33.62 ± 6.73	†⁑	34.37 ± 1.06		T	0.000	0.165
	Total	*77*	36.15 ± 6.58	35.54 ± 6.82	†	35.04 ± 6.63	†	35.56 ± 0.74		G x T	0.085	0.033

Data are expressed as means ± standard deviations for the placebo (PLA) and probiotics (PRO) groups. Data were analyzed using a multivariate and univariate General Linear Model with repeated measures. P-levels, with partial ETA squared (η_p^2), are listed for between-subject group (G) and univariate within-subject (Greenhouse-Geisser) time (T), and group x time (G x T) effects. Multivariate Wilk's Lambda showed significant time ($p < 0.001$, $\eta_p^2 = 0.212$), but no group x time ($p = 0.207$, $\eta_p^2 = 0.036$) within-subject effects. Pairwise comparisons, with LSD confidence interval adjustment, for simple main effects are indicated by the following superscripts: difference from baseline value, † = p<0.05 (‡ = p>0.05 to p<0.10); and between groups, * = p<0.05 (⁑ = p>0.05 to p<0.10). η_p^2 = effect size where values of 0.01 - 0.05 = small, 0.06 - 0.13 = medium, and >0.14 = large.

Table S5. Visceral adipose tissue analysis.

Variable	Group	N	Weeks 0	Weeks 6		Weeks 12		Mean (SEM)		Effect	p-Level	η_p^2
Android Fat	PLA	*39*	37.94 ± 5.43	37.19 ± 5.87		36.69 ± 6.34	‡	37.27 ± 0.97		G	0.063	0.045
(%)	PRO	*38*	36.01 ± 6.68	33.89 ± 6.89	†*	34.09 ± 7.18	†⁑	34.66 ± 0.98	⁑	T	0.000	0.106
	Total	*77*	36.98 ± 6.12	35.56 ± 6.56	†	35.41 ± 6.85	†	35.97 ± 0.69		G x T	0.256	0.018
Gynoid Fat	PLA	*39*	38.64 ± 7.03	38.16 ± 7.21	‡	38.13 ± 6.90		38.31 ± 1.14		G	0.265	0.017
(%)	PRO	*38*	37.26 ± 7.65	36.46 ± 7.36	†	35.74 ± 7.16	†	36.49 ± 1.16		T	0.000	0.111
	Total	*77*	37.96 ± 7.33	37.32 ± 7.29	†	36.95 ± 7.09	†	37.40 ± 0.81		G x T	0.100	0.031
VAT	PLA	*39*	519 ± 222	510 ± 223		504 ± 220		511 ± 33		G	0.251	0.017
(g)	PRO	*38*	476 ± 206	450 ± 192	†	447 ± 184	‡	457 ± 33		T	0.054	0.040
	Total	*77*	497 ± 214	480 ± 209	†	476 ± 204	†	484 ± 23		G x T	0.553	0.007
VAT Volume	PLA	*39*	561 ± 240	551 ± 241		545 ± 238		552 ± 35		G	0.252	0.017
(cm^3)	PRO	*38*	515 ± 223	486 ± 207	†	483 ± 199	‡	495 ± 36		T	0.054	0.040
	Total	*77*	538 ± 231	519 ± 226	†	515 ± 221	†	524 ± 25		G x T	0.553	0.007
VAT Area	PLA	*39*	107.6 ± 46.0	105.8 ± 46.3		104.7 ± 45.8		106.0 ± 6.8		G	0.250	0.018
(cm^2)	PRO	*38*	98.7 ± 42.8	93.2 ± 39.8	†	92.7 ± 38.2	‡	94.9 ± 6.8		T	0.054	0.040
	Total	*77*	103.2 ± 44.4	99.6 ± 43.4	†	98.8 ± 42.4	†	100.5 ± 4.8		G x T	0.556	0.007

Data are expressed as means, standard deviations for the placebo (PLA) and plant probiotic (PRO) groups. Data were analyzed using a multivariate and univariate General Linear Model with repeated measures. P-levels, with partial ETA squared (η_p^2), are listed for between-subject group (G) and univariate within-subject (Greenhouse-Geisser) time (T), and group x time (G x T) effects. Multivariate Wilk's Lambda showed significant time ($p < 0.003$, $\eta_p^2 = 0.088$), but no group x time ($p = 0.405$, $\eta_p^2 = 0.035$) within-subject effects. VAT = visceral adipose tissue. Pairwise comparisons, with LSD confidence interval adjustment, for simple main effects are indicated by the following superscripts: difference from baseline value, † = p<0.05 (‡ = p>0.05 to p<0.10); and between groups, * = p<0.05 (⁑ = p>0.05 to p<0.10). η_p^2 = effect size where values of 0.01 - 0.05 = small, 0.06 - 0.13 = medium, and >0.14 = large.

Table S6. Bone related variables.

Variable	Group	N	Weeks 0	Weeks 6	Weeks 12	Mean (SEM)	Effect	p-Level	η_p^2
Bone Mineral Content (g)	PLA	*39*	1866 ± 374	1857 ± 373	1852 ± 373	1859 ± 51	G	0.574	0.004
	PRO	*38*	1808 ± 256	1803 ± 253	1788 ± 242 †	1800 ± 52	T	0.011	0.063
	Total	*77*	1837 ± 320	1831 ± 318	1821 ± 315 †	1829 ± 36	G x T	0.615	0.006
Bone Mineral Area (cm^2)	PLA	*39*	1850 ± 216	1839 ± 211 ‡	1837 ± 216	1842 ± 29	G	0.083	0.040
	PRO	*38*	1840 ± 158	1826 ± 154 †	1807 ± 136 †	1825 ± 30	T	0.001	0.101
	Total	*77*	1845 ± 188	1833 ± 184 †	1822 ± 180 †	1833 ± 21	G x T	0.171	0.024
Bone Mineral Density (cm^2)	PLA	*39*	1.0001 ± 0.0986	1.0015 ± 0.0982	0.9997 ± 0.0973	1.0005 ± 0.0136	G	0.686	0.002
	PRO	*38*	0.9791 ± 0.0724	0.9838 ± 0.0686	0.9861 ± 0.0729 †	0.9830 ± 0.0138	T	0.219	0.020
	Total	*77*	0.9897 ± 0.0868	0.9928 ± 0.0848	0.9930 ± 0.0858	0.9917 ± 0.0097	G x T	0.225	0.020

Data are expressed as means, standard deviations for the placebo (PLA) and plant probiotic (PRO) groups. Data were analyzed using a multivariate and univariate General Linear Model with repeated measures. P-levels, with partial ETA squared (η_p^2), are listed for between-subject group (G) and univariate within-subject (Greenhouse-Geisser) time (T), and group x time (G x T) effects. Multivariate Wilk's Lambda showed a significant time ($p = 0.01$, $\eta_p^2 = 0.055$), but no group x time ($p = 0.558$, $\eta_p^2 = 0.016$) within-subject effects. Pairwise comparisons, with LSD confidence interval adjustment, for simple main effects are indicated by the following superscripts: difference from baseline value, † = p<0.05 (‡ = p>0.05 to p<0.10); and between groups, * = p<0.05 (⁑ = p>0.05 to p<0.10). η_p^2 = effect size where values of 0.01 - 0.05 = small, 0.06 - 0.13 = medium, and >0.14 = large.

Table S7. Resting energy expenditure and substrate utilization.

Variable	Group	N	Weeks 0	6	12	Mean (SEM)	Effect	p-Level	η_p^2
Resting Energy Expenditure	PLA	*39*	1475 ± 284	1419 ± 211	1442 ± 240	1445 ± 35	G	0.762	0.001
(kcals/d)	PRO	*38*	1457 ± 248	1473 ± 263	1451 ± 273	1460 ± 36	T	0.671	0.005
	Total	*77*	1466 ± 265	1446 ± 238	1446 ± 255	1453 ± 25	G x T	0.366	0.013
Respiratory Quotient	PLA	*39*	0.84 ± 0.08	0.82 ± 0.06	0.85 ± 0.10	0.84 ± 0.01	G	0.784	0.001
	PRO	*38*	0.84 ± 0.06	0.83 ± 0.08	0.83 ± 0.07	0.83 ± 0.01	T	0.244	0.019
	Total	*77*	0.84 ± 0.07	0.82 ± 0.07	0.84 ± 0.08	0.84 ± 0.01	G x T	0.375	0.013
Carbohydrate Oxidation	PLA	*39*	44.4 ± 24.2	40.4 ± 19.3	45.8 ± 17.9	43.5 ± 2.2	G	0.842	0.001
(%)	PRO	*38*	48.0 ± 18.5	40.7 ± 19.0	43.8 ± 22.8	44.2 ± 2.2	T	0.163	0.024
	Total	*77*	46.1 ± 21.5	40.6 ± 19.1 ‡	44.8 ± 20.3	43.9 ± 1.5	G x T	0.648	0.006
Fat Oxidation	PLA	*39*	55.6 ± 24.2	59.6 ± 19.3	54.2 ± 17.9	56.5 ± 2.2	G	0.842	0.001
(%)	PRO	*38*	52.0 ± 18.5	59.3 ± 19.0	56.2 ± 22.8	55.8 ± 2.2	T	0.163	0.024
	Total	*77*	53.9 ± 21.5	59.4 ± 19.1 ‡	55.2 ± 20.3	56.1 ± 1.5	G x T	0.648	0.006

Data are expressed as means, standard deviations for the placebo (PLA) and plant probiotic (PRO) groups. Data were analyzed using a multivariate and univariate General Linear Model with repeated measures. P-levels, with partial ETA squared (η_p^2), are listed for between-subject group (G) and univariate within-subject (Greenhouse-Geisser) time (T), and group x time (G x T) effects. Multivariate Wilk's Lambda showed no significant time ($p = 0.441$, $\eta_p^2 = 0.019$) or group x time ($p = 0.076$, $\eta_p^2 = 0.784$) within-subject effects. Pairwise comparisons, with LSD confidence interval adjustment, for simple main effects are indicated by the following superscripts: difference from baseline value, † = p<0.05 (‡ = p>0.05 to p<0.10); and between groups, * = p<0.05 (⁑ = p>0.05 to p<0.10). η_p^2 = effect size where values of 0.01 - 0.05 = small, 0.06 - 0.13 = medium, and >0.14 = large.

Table S8. Oxygen Uptake and Steps per Day

Variable	Group	N	Weeks 0		Weeks 6			Weeks 12			Mean (SEM)	Effect	p-Level	η_p^2
Peak Oxygen Uptake	PLA	*39*	2.261	± 0.66	2.314	± 0.69		2.259	± 0.65		2.278 ± 0.10	G	0.574	0.004
(L/min)	PRO	*38*	2.364	± 0.66	2.236	± 0.74	†	2.190	± 0.61	†	2.263 ± 0.11	T	0.062	0.038
	Total	*77*	2.311	± 0.66	2.276	± 0.71		2.225	± 0.63	†	2.270 ± 0.07	G x T	0.026	0.050
Peak Oxygen Uptake	PLA	*39*	28.36	± 7.14	29.88	± 7.64	‡	28.86	± 7.17		29.03 ± 1.10	G	0.083	0.040
(mL/kg/min)	PRO	*38*	29.60	± 6.85	29.61	± 9.13		28.71	± 6.73		29.31 ± 1.13	T	0.224	0.020
	Total	*77*	28.96	± 6.98	29.75	± 8.34		28.79	± 6.91		29.17 ± 0.79	G x T	0.340	0.014
Steps per Day	PLA	*39*	7006	± 2473	9059	± 2004	†	9050	± 1963	†	8372 ± 306	G	0.686	0.002
	PRO	*38*	7397	± 2568	9741	± 2196	†	9810	± 2204	†	8983 ± 314	T	0.000	0.445
	Total	*77*	7197	± 2511	9391	± 2114	†	9420	± 2105	†	8677 ± 219	G x T	0.648	0.005

Data are expressed as means, standard deviations for the placebo (PLA) and plant probiotic (PRO) groups. Data were analyzed using a multivariate and univariate General Linear Model with repeated measures. P-levels, with partial ETA squared (η_p^2), are listed for between-subject group (G) and univariate within-subject (Greenhouse-Geisser) time (T), and group x time (G x T) effects. Multivariate Wilk's Lambda showed a significant time ($p < 0.01$, $\eta_p^2 = 0.301$), but no group x time ($p = 0.095$, $\eta_p^2 = 0.036$) within-subject effects. Pairwise comparisons, with LSD confidence interval adjustment, for simple main effects are indicated by the following superscripts: difference from baseline value, † = p<0.05 (‡ = p>0.05 to p<0.10); and between groups, * = p<0.05 (⁑ = p>0.05 to p<0.10). η_p^2 = effect size where values of 0.01 - 0.05 = small, 0.06 - 0.13 = medium, and >0.14 = large.

Table S9. Complete Blood Count.

Variable	Group	N	Weeks 0	Weeks 6	Weeks 12	Mean (SEM)	Effect	p-Level	η_p^2
White Blood Cells	PLA	*39*	6.21 ± 2.46	5.54 ± 1.43 †	5.89 ± 0.41	5.88 ± 0.24	Group	0.974	0.000
[K/μL]	PRO	*37*	5.92 ± 1.26	5.65 ± 1.36	6.03 ± 0.46	5.87 ± 0.25	Time	0.010	0.064
	Total	*76*	6.07 ± 1.96	5.59 ± 1.39 †	5.95 ± 0.44	5.87 ± 0.17	G x T	0.306	0.016
Red Blood Cells	PLA	*39*	4.70 ± 0.41	4.67 ± 0.47	4.67 ± 0.47	4.68 ± 0.07	Group	0.095	0.037
[M/μL]	PRO	*37*	4.85 ± 0.46	4.84 ± 0.40	4.84 ± 0.40 ⁑	4.85 ± 0.07	Time	0.729	0.004
	Total	*76*	4.77 ± 0.44	4.75 ± 0.45	4.75 ± 0.45	4.77 ± 0.05	G x T	0.892	0.001
Hemoglobin	PLA	*39*	13.67 ± 1.48	13.71 ± 1.59	13.65 ± 1.49	13.68 ± 0.57	Group	0.142	0.029
[g/dL]	PRO	*37*	14.14 ± 1.40	14.16 ± 1.37	14.15 ± 1.30	14.15 ± 0.59	Time	0.863	0.002
	Total	*76*	13.90 ± 1.45	13.93 ± 1.49	13.89 ± 1.41	13.91 ± 0.16	G x T	0.908	0.001
Hematocrit	PLA	*39*	41.32 ± 3.92	41.44 ± 4.18	41.31 ± 3.91	41.36 ± 0.57	Group	0.153	0.027
[%]	PRO	*37*	42.64 ± 3.82	42.55 ± 3.50	42.43 ± 3.13	42.54 ± 0.59	Time	0.848	0.002
	Total	*76*	41.96 ± 3.90	41.98 ± 3.88	41.86 ± 3.57	41.95 ± 0.41	G x T	0.881	0.002
Mean Corpuscular Volume	PLA	*39*	88.04 ± 5.90	88.54 ± 5.90 ‡	88.72 ± 5.98 ‡	88.44 ± 0.85	Group	0.695	0.002
[fL]	PRO	*37*	88.02 ± 4.67	88.01 ± 4.67	87.83 ± 4.71	87.96 ± 0.87	Time	0.520	0.008
	Total	*76*	88.03 ± 5.30	88.28 ± 5.30	88.29 ± 5.38	88.20 ± 0.61	G x T	0.226	0.020
Mean Corpuscular Hemoglobin	PLA	*39*	29.12 ± 2.57	29.30 ± 2.77	29.31 ± 2.65	29.24 ± 0.36	Group	0.989	0.000
[pg]	PRO	*37*	29.18 ± 1.77	29.26 ± 1.79	29.27 ± 1.92	29.24 ± 0.37 ⁑	Time	0.199	0.022
	Total	*76*	29.15 ± 2.21	29.28 ± 2.33 ‡	29.29 ± 2.31	29.24 ± 0.26	G x T	0.781	0.003
Mean Corpuscular Hemoglobin Concentration	PLA	*39*	33.03 ± 1.14	33.04 ± 1.26 ‡	32.99 ± 1.14	33.02 ± 0.15	Group	0.334	0.013
[g/dL]	PRO	*37*	33.15 ± 0.86	33.24 ± 0.96	33.31 ± 0.88	33.23 ± 0.16	Time	0.726	0.004
	Total	*76*	33.09 ± 1.01	33.14 ± 1.12	33.14 ± 1.03	33.13 ± 0.11	G x T	0.475	0.010
Red Blood Cell Distribution Width	PLA	*39*	12.90 ± 0.95	12.94 ± 0.97	12.86 ± 1.11	12.90 ± 0.14	Group	0.430	0.008
[%]	PRO	*37*	12.69 ± 0.86	12.76 ± 0.84	12.76 ± 0.88	12.74 ± 0.15	Time	0.554	0.007
	Total	*76*	12.80 ± 0.91	12.85 ± 0.91	12.81 ± 1.00	12.82 ± 0.10	G x T	0.583	0.006
Neutrophils	PLA	*39*	59.01 ± 9.58	57.07 ± 7.41	56.71 ± 7.99	57.60 ± 1.05	Group	0.185	0.024
[%]	PRO	*37*	55.65 ± 7.62 ⁑	54.79 ± 6.45	56.33 ± 7.69	55.59 ± 1.08	Time	0.271	0.018
	Total	*76*	57.37 ± 8.79	55.96 ± 7.01	56.52 ± 7.79	56.59 ± 0.75	G x T	0.221	0.020
Lymphocytes	PLA	*39*	30.60 ± 8.71	31.75 ± 6.62	32.21 ± 7.20	31.52 ± 0.99	Group	0.275	0.016
[%]	PRO	*37*	33.17 ± 7.25	33.74 ± 6.20	32.35 ± 7.39	33.09 ± 1.02	Time	0.520	0.009
	Total	*76*	31.85 ± 8.08	32.72 ± 6.45	32.28 ± 7.25	32.31 ± 0.71	G x T	0.248	0.019
Neutrophil : Lymphocyte Ratio	PLA	*39*	2.25 ± 1.29	1.93 ± 0.69 †	1.93 ± 0.84 †	2.04 ± 0.11	Group	0.127	0.031
	PRO	*37*	1.81 ± 0.66 ⁑	1.72 ± 0.57	1.88 ± 0.65	1.80 ± 0.11	Time	0.120	0.029
	Total	*76*	2.03 ± 1.05	1.83 ± 0.64 ‡	1.91 ± 0.75	1.92 ± 0.08	G x T	0.142	0.027
Monocytes	PLA	*39*	7.08 ± 2.00	7.45 ± 2.18	7.29 ± 2.24	7.27 ± 0.27	Group	0.444	0.008
[%]	PRO	*37*	7.54 ± 1.69	7.58 ± 1.45	7.59 ± 1.38	7.57 ± 0.27	Time	0.463	0.010
	Total	*76*	7.30 ± 1.86	7.51 ± 1.85	7.43 ± 1.86	7.42 ± 0.19	G x T	0.623	0.006
Eosinophils	PLA	*39*	2.40 ± 1.74	2.77 ± 2.13 †	2.75 ± 1.71 ‡	2.64 ± 0.26	Group	0.805	0.001
[%]	PRO	*37*	2.64 ± 1.73	2.85 ± 1.52	2.71 ± 1.54	2.73 ± 0.26	Time	0.089	0.033
	Total	*76*	2.52 ± 1.73	2.81 ± 1.85 †	2.73 ± 1.62	2.69 ± 0.18	G x T	0.585	0.007
Basophils	PLA	*39*	0.64 ± 0.31	0.73 ± 0.33	0.76 ± 0.31	0.71 ± 0.05	Group	0.319	0.013
[%]	PRO	*37*	0.78 ± 0.40 ⁑	0.79 ± 0.33	0.78 ± 0.34	0.78 ± 0.05	Time	0.175	0.023
	Total	*76*	0.71 ± 0.36	0.76 ± 0.33	0.77 ± 0.32	0.75 ± 0.19	G x T	0.158	0.025
Granulocytes	PLA	*39*	0.273 ± 0.143	0.271 ± 0.249	0.271 ± 0.249	0.258 ± 0.022	Group	0.679	0.002
[%]	PRO	*37*	0.238 ± 0.180	0.242 ± 0.140	0.242 ± 0.140	0.245 ± 0.023	Time	0.849	0.002
	Total	*76*	0.256 ± 0.162	0.257 ± 0.202	0.257 ± 0.202	0.251 ± 0.016	G x T	0.521	0.009
PLAatelets	PLA	*39*	269.5 ± 71.0	263.5 ± 66.0	262.6 ± 63.2	265.2 ± 10.5	Group	0.677	0.002
[K/μL]	PRO	*37*	269.6 ± 71.1	269.3 ± 67.2	275.5 ± 71.0	271.5 ± 10.8	Time	0.664	0.005
	Total	*76*	269.6 ± 70.6	266.3 ± 66.2	268.9 ± 67.0	268.3 ± 7.5	G x T	0.239	0.019

Data are expressed as means, standard deviations for the placebo (PLA) and plant probiotic (PRO) groups. Data were analyzed using a multivariate and univariate General Linear Model with repeated measures. P-levels, with partial ETA squared (η_p^2), are listed for between-subject group (G) and univariate within-subject (Greenhouse-Geisser) time (T), and group x time (G x T) effects. Multivariate Wilk's Lambda showed no significant time ($p = 0.112$, $\eta_p^2 = 0.139$ or group x time ($p = 0.886$, $\eta_p^2 = 0.078$) within-subject effects. Pairwise comparisons, with LSD confidence interval adjustment, for simple main effects are indicated by the following superscripts: difference from baseline value, † = $p<0.05$ (‡ = $p>0.05$ to $p<0.10$); and between groups, * = $p<0.05$ (⁑ = $p>0.05$ to $p<0.10$). η_p^2 = effect size where values of 0.01 - 0.05 = small, 0.06 - 0.13 = medium, and >0.14 = large.

Table S10. Serum chemistry markers.

Variable	Group	N	Weeks 0	Weeks 6	Weeks 12	Mean (SEM)	Effect	p-Level	η_p^2
Glucose	PLA	*37*	96.97 ± 9.89	97.88 ± 8.20	96.36 ± 0.40	97.07 ± 1.11	Group	<.001	0.145
(mg/dL)	PRO	*37*	91.95 ± 7.02 *	91.91 ± 6.46 *	90.95 ± 0.30 *	91.60 ± 1.11 *	Time	0.305	0.016
	Total	*74*	94.46 ± 8.89	94.89 ± 7.92	93.65 ± 0.36	94.34 ± 0.78	G x T	0.828	0.002
Glycoslyated Hemoglobin	PLA	*37*	5.48 ± 0.40	5.45 ± 0.37 ‡	5.45 ± 0.37	5.45 ± 0.05	Group	0.068	0.046
(%)	PRO	*37*	5.31 ± 0.30	5.34 ± 0.23	5.34 ± 0.23	5.32 ± 0.05 ⁑	Time	0.411	0.012
	Total	*74*	5.39 ± 0.36	5.39 ± 0.31	5.39 ± 0.31	5.39 ± 0.04	G x T	0.397	0.012
Blood Urea Nitrogen	PLA	*37*	12.57 ± 2.95	12.92 ± 4.20	12.39 ± 3.47	12.62 ± 0.03	Group	0.043	0.056
(mg/dL)	PRO	*37*	14.22 ± 4.24 ⁑	14.20 ± 3.73	14.03 ± 3.86	14.15 ± 0.03 *	Time	0.685	0.005
	Total	*74*	13.39 ± 3.72	13.56 ± 4.00	13.21 ± 3.74	13.39 ± 0.37	G x T	0.876	0.002
Creatinine	PLA	*37*	0.84 ± 0.20	0.88 ± 0.22 †	0.86 ± 0.19	0.86 ± 0.03	Group	0.107	0.036
(mg/dL)	PRO	*37*	0.95 ± 0.23 *	0.95 ± 0.21	0.91 ± 0.19 †	0.94 ± 0.03	Time	0.011	0.061
	Total	*74*	0.89 ± 0.22	0.92 ± 0.22 †	0.89 ± 0.19	0.90 ± 0.02	G x T	0.012	0.059
BUN-Creatinine Ratio	PLA	*37*	15.65 ± 4.74	14.80 ± 4.74	14.75 ± 4.39	15.06 ± 0.64	Group	0.513	0.006
	PRO	*37*	15.49 ± 4.97	15.45 ± 4.97	16.05 ± 5.33	15.66 ± 0.64	Time	0.652	0.005
	Total	*74*	15.57 ± 4.82	15.12 ± 4.82	15.40 ± 4.89	15.36 ± 0.46	G x T	0.348	0.014
Total Proteins	PLA	*37*	7.03 ± 0.47	6.95 ± 0.48	6.98 ± 0.59	6.99 ± 0.07	Group	0.311	0.014
(g/dL)	PRO	*37*	7.06 ± 0.48	7.10 ± 0.44	7.11 ± 0.45	7.09 ± 0.07 ⁑	Time	0.848	0.002
	Total	*74*	7.05 ± 0.47	7.03 ± 0.46	7.04 ± 0.52	7.04 ± 0.05	G x T	0.365	0.014
Albumin	PLA	*37*	4.62 ± 0.27	4.62 ± 0.27	4.58 ± 0.30	4.60 ± 0.04	Group	0.358	0.012
(g/dL)	PRO	*37*	4.65 ± 0.32	4.70 ± 0.31	4.61 ± 0.30	4.66 ± 0.04	Time	0.126	0.029
	Total	*74*	4.64 ± 0.30	4.66 ± 0.29	4.59 ± 0.30	4.63 ± 0.03	G x T	0.654	0.006
Globulin	PLA	*37*	2.41 ± 0.38	2.34 ± 0.37 †	2.40 ± 0.42	2.38 ± 0.06	Group	0.529	0.006
(g/dL)	PRO	*37*	2.41 ± 0.35	2.39 ± 0.37	2.49 ± 0.35 ‡	2.43 ± 0.06	Time	0.012	0.062
	Total	*74*	2.41 ± 0.36	2.36 ± 0.37 ‡	2.45 ± 0.38	2.41 ± 0.04	G x T	0.265	0.018
Albumin-Globulin Ratio	PLA	*37*	1.97 ± 0.33	2.03 ± 0.34	1.97 ± 0.35	1.99 ± 0.05	Group	0.681	0.002
	PRO	*37*	1.97 ± 0.33	2.03 ± 0.45	1.88 ± 0.28 †	1.96 ± 0.05	Time	0.005	0.074
	Total	*74*	1.97 ± 0.33	2.03 ± 0.40 †	1.92 ± 0.32	1.98 ± 0.04	G x T	0.281	0.017
Bilirubin	PLA	*37*	0.58 ± 0.30	0.61 ± 0.34	0.57 ± 0.34	0.58 ± 0.05	Group	0.562	0.005
(mg/dL)	PRO	*37*	0.54 ± 0.25	0.54 ± 0.27	0.55 ± 0.37	0.54 ± 0.05	Time	0.626	0.006
	Total	*74*	0.56 ± 0.28	0.58 ± 0.30	0.56 ± 0.35	0.56 ± 0.03	G x T	0.586	0.007
Alkaline Phosphatase	PLA	*37*	69.65 ± 18.30	70.24 ± 19.64	70.24 ± 16.60	70.04 ± 3.06	Group	0.972	0.000
(U/L)	PRO	*37*	70.08 ± 21.67	69.51 ± 22.25	70.09 ± 20.23	69.89 ± 3.06	Time	0.966	0.000
	Total	*74*	69.86 ± 19.92	69.88 ± 20.84	70.16 ± 18.38	69.97 ± 2.16	G x T	0.908	0.001
Aspartate Aminotransferase	PLA	*37*	22.14 ± 8.97	20.46 ± 6.84 ‡	21.00 ± 6.66	21.20 ± 1.12	Group	0.341	0.013
(U/L)	PRO	*37*	22.41 ± 6.28	20.55 ± 5.70 †	25.19 ± 13.26 ‡⁑	22.72 ± 1.12	Time	0.040	0.049
	Total	*74*	22.27 ± 7.69	20.51 ± 6.25 †	23.09 ± 10.63	21.96 ± 0.79	G x T	0.078	0.038
Alaninie Aminotransferase	PLA	*37*	25.27 ± 19.08	25.27 ± 19.08	23.96 ± 21.10	69.89 ± 3.06	Group	0.727	0.002
(U/L)	PRO	*37*	24.08 ± 14.20	24.08 ± 14.20	21.22 ± 9.78	21.20 ± 1.12	Time	0.453	0.011
	Total	*74*	24.68 ± 16.72	24.68 ± 16.72	22.59 ± 16.39	0.56 ± 0.03	G x T	0.636	0.006
Estimated Glomular Filtration Rate	PLA	*37*	102.06 ± 20.49	99.07 ± 22.30 †	100.38 ± 19.50	24.34 ± 2.32	Group	0.514	0.006
(ml/min/1.73)	PRO	*37*	96.93 ± 21.45	95.08 ± 19.92	100.57 ± 18.25 †	23.19 ± 2.32	Time	0.016	0.058
	Total	*74*	99.49 ± 20.99	97.07 ± 21.09 †	100.47 ± 18.76	23.77 ± 1.64	G x T	0.066	0.038

Data are expressed as means ± standard deviations for the placebo (PLA) and probiotics (PRO) groups. Data were analyzed using a multivariate and univariate General Linear Model with repeated measures. P-levels, with partial ETA squared (η_p^2), are listed for between-subject group (G) and univariate within-subject (Greenhouse-Geisser) time (T), and group x time (G x T) effects. Multivariate Wilk's Lambda showed no significant time ($p = 0.060$, $\eta_p^2 = 0.129$) or group x time ($p = 0.090$, $\eta_p^2 = 0.123$) within-subject effects. Pairwise comparisons, with LSD confidence interval adjustment, for simple main effects are indicated by the following superscripts: difference from baseline value, † = $p<0.05$ (‡ = $p>0.05$ to $p<0.10$); and between groups, * = $p<0.05$ (⁑ = $p>0.05$ to $p<0.10$). η_p^2 = effect size where values of 0.01 - 0.05 = small, 0.06 - 0.13 = medium, and >0.14 = large.

Table S11. Serum lipid results.

Variable	Group	N	Weeks 0	6	12	Mean (SEM)	Effect	p-Level	η_p^2
Total Cholesterol	PLA	*39*	178.3 ± 38.1	176.4 ± 34.1	178.9 ± 61.2	177.9 ± 5.2	Group	0.329	0.013
[mg/dL]	PRO	*37*	185.9 ± 32.5	183.1 ± 36.7	186.7 ± 39.2	185.2 ± 5.4	Time	0.478	0.010
	Total	*76*	182.0 ± 35.5	179.7 ± 35.3	182.7 ± 51.9	181.6 ± 3.7	G x T	0.975	0.000
Triglycerides	PLA	*39*	109.4 ± 61.2	106.8 ± 64.0	106.8 ± 64.0	106.9 ± 7.9	Group	0.339	0.012
[mg/dL]	PRO	*37*	95.2 ± 39.2	103.1 ± 53.7	103.1 ± 53.7	95.9 ± 8.2	Time	0.131	0.028
	Total	*76*	102.4 ± 51.9	105.0 ± 58.8	105.0 ± 58.8	101.4 ± 5.7	G x T	0.280	0.017
HDL Cholesterol	PLA	*39*	51.0 ± 16.2	51.6 ± 19.0	51.8 ± 16.9	51.5 ± 4.7	Group	0.467	0.007
[mg/dL]	PRO	*37*	54.1 ± 15.9	52.9 ± 16.1	55.6 ± 18.1	54.2 ± 4.8	Time	0.322	0.015
	Total	*76*	52.5 ± 16.0	52.2 ± 17.6	53.6 ± 17.5	52.8 ± 1.9	G x T	0.448	0.010
LDL Cholesterol	PLA	*39*	106.6 ± 31.5	104.1 ± 30.8	106.5 ± 29.6	105.8 ± 4.7	Group	0.372	0.011
[mg/dL]	PRO	*37*	112.6 ± 28.8	111.8 ± 34.0	110.9 ± 32.6	111.7 ± 4.8	Time	0.762	0.004
	Total	*76*	109.5 ± 30.2	107.9 ± 32.4	108.6 ± 30.9	108.7 ± 3.3	G x T	0.766	0.003
Non-HDL Cholesterol	PLA	*39*	127.3 ± 36.5	124.5 ± 36.5	127.1 ± 34.6	126.3 ± 5.3	Group	0.532	0.005
[mg/dL]	PRO	*37*	131.8 ± 31.2	130.3 ± 31.2	131.1 ± 36.4	131.1 ± 5.4	Time	0.648	0.006
	Total	*76*	129.5 ± 33.9	127.3 ± 33.9	129.1 ± 35.3	128.7 ± 3.8	G x T	0.917	0.001
VLDL Cholesterol	PLA	*39*	20.67 ± 7.71	20.33 ± 8.28	20.65 ± 8.39	20.55 ± 1.06	Group	0.424	0.009
[mg/dL]	PRO	*37*	19.22 ± 5.16	18.55 ± 4.78	20.22 ± 6.93	19.33 ± 1.08	Time	0.144	0.026
	Total	*76*	19.96 ± 6.59	19.47 ± 6.82	20.44 ± 7.67	19.94 ± 0.76	G x T	0.368	0.013
LDL:HDL Ratio	PLA	*39*	2.28 ± 0.93	2.30 ± 1.10	2.31 ± 0.99	2.30 ± 0.16	Group	0.963	0.000
	PRO	*37*	2.30 ± 0.98	2.35 ± 1.07	2.28 ± 1.14	2.31 ± 0.16	Time	0.843	0.002
	Total	*76*	2.29 ± 0.95	2.32 ± 1.08	2.29 ± 1.06	2.30 ± 0.11	G x T	0.798	0.003
Total Cholesterol:HDL Ratio	PLA	*39*	3.73 ± 1.13	3.76 ± 1.34	3.73 ± 1.25	3.74 ± 0.19	Group	0.933	0.000
	PRO	*37*	3.71 ± 1.17	3.74 ± 1.23	3.71 ± 1.38	3.72 ± 0.20	Time	0.841	0.002
	Total	*76*	3.72 ± 1.14	3.75 ± 1.28	3.72 ± 1.31	3.73 ± 0.14	G x T	0.996	0.000

Data are expressed as means, standard deviations for the placebo (PLA) and plant probiotic (PRO) groups. Data were analyzed using a multivariate and univariate General Linear Model with repeated measures. P-levels, with partial ETA squared (η_p^2), are listed for between-subject group (G) and univariate within-subject (Greenhouse-Geisser) time (T), and group x time (G x T) effects. Multivariate Wilk's Lambda showed no significant time ($p = 0.083$, $\eta_p^2 = 0.072$) or group x time ($p = 0.338$, $\eta_p^2 = 0.052$) within-subject effects. Pairwise comparisons, with LSD confidence interval adjustment, for simple main effects are indicated by the following superscripts: difference from baseline value, † = p<0.05 (‡ = p>0.05 to p<0.10); and between groups, * = p<0.05 (⁑ = p>0.05 to p<0.10). η_p^2 = effect size where values of 0.01 - 0.05 = small, 0.06 - 0.13 = medium, and >0.14 = large.

Table 12. Serum electrolytes.

Variable	Group	N	Weeks 0	6	12	Mean (SEM)	Effect	p-Level	η_p^2
Sodium	PLA	*39*	140.5 ± 2.0	140.8 ± 1.8	140.5 ± 1.9	140.6 ± 0.3	G	0.446	0.008
(meq/L)	PRO	*37*	140.8 ± 2.5	141.0 ± 2.4	140.8 ± 2.2	140.9 ± 0.3	T	0.624	0.006
	Total	*76*	140.7 ± 2.2	140.9 ± 2.1	140.7 ± 2.0	140.7 ± 0.2	G x T	0.990	0.000
Potassium	PLA	*39*	4.37 ± 0.39	4.41 ± 0.31	4.42 ± 0.36	4.40 ± 0.04	G	0.576	0.004
(meq/L)	PRO	*37*	4.45 ± 0.33	4.44 ± 0.41	4.43 ± 0.34	4.44 ± 0.04	T	0.949	0.001
	Total	*76*	4.41 ± 0.36	4.42 ± 0.36	4.43 ± 0.35	4.42 ± 0.03	G x T	0.759	0.004
Chloride	PLA	*39*	103.56 ± 1.65	103.68 ± 1.98	103.87 ± 2.04	103.70 ± 0.25	G	0.741	0.001
(meq/L)	PRO	*37*	103.78 ± 2.23	103.91 ± 2.24	103.78 ± 1.73	103.82 ± 0.26	T	0.815	0.003
	Total	*76*	103.67 ± 1.94	103.79 ± 2.10	103.82 ± 1.89	103.76 ± 0.18	G x T	0.763	0.004
Calcium	PLA	*39*	9.71 ± 0.42	9.69 ± 0.42	9.69 ± 0.38	9.69 ± 0.05	G	0.913	0.000
(meq/L)	PRO	*37*	9.67 ± 0.38	9.73 ± 0.38	9.66 ± 0.32	9.69 ± 0.05	T	0.723	0.004
	Total	*76*	9.69 ± 0.40	9.71 ± 0.40	9.67 ± 0.35	9.69 ± 0.03	G x T	0.583	0.007
Carbon Dioxide	PLA	*39*	24.72 ± 1.95	24.83 ± 2.21	24.71 ± 2.21	24.75 ± 0.23	G	0.412	0.009
(mg/dL)	PRO	*37*	24.27 ± 1.76	24.88 ± 1.84	24.31 ± 1.84	24.48 ± 0.23	T	0.239	0.019
	Total	*76*	24.50 ± 1.86	24.86 ± 2.02	24.51 ± 2.02	24.62 ± 0.16	G x T	0.523	0.009

Data are expressed as means, standard deviations for the placebo (PLA) and plant probiotic (PRO) groups. Data were analyzed using a multivariate and univariate General Linear Model with repeated measures. P-levels, with partial ETA squared (η_p^2), are listed for between-subject group (G) and univariate within-subject (Greenhouse-Geisser) time (T), and group x time (G x T) effects. Multivariate Wilk's Lambda showed no significant time ($p < 0.895$, $\eta_p^2 = 0.017$) or group x time ($p = 0.803$, $\eta_p^2 = 0.021$) within-subject effects. Pairwise comparisons, with LSD confidence interval adjustment, for simple main effects are indicated by the following superscripts: difference from baseline value, † = p<0.05 (‡ = p>0.05 to p<0.10); and between groups, * = p<0.05 (⁑ = p>0.05 to p<0.10). η_p^2 = effect size where values of 0.01 - 0.05 = small, 0.06 - 0.13 = medium, and >0.14 = large.

Table S13. Quality of Life ratings.

Symptom	Week	Group	Excellent	Very Good	Good	Fair	Poor	p-level
Health	0	PLA	0	13	24	2	0	0.263
		PRO	3	12	18	2	0	
		Total	3	28	42	4	0	
	6	PLA	1	20	17	1	0	0.169
		PRO	6	16	16	0	0	
		Total	7	36	33	1	0	
	12	PLA	4	17	16	2	0	0.385
		PRO	7	20	9	2	0	
		Total	11	37	25	4	0	

Symptom	Week	Group	Much Better	Somewhat Better	About the Same	Somewhat Worse	Much Worse	*p* -level
Health Compared to One Year Ago	0	PLA	0	8	19	12	0	0.258
		PRO	3	8	19	7	1	
		Total	3	16	38	19	0	
	6	PLA	5	15	14	5	0	0.718
		PRO	8	15	12	3	0	
		Total	13	30	26	8	0	
	12	PLA	3	21	10	5	0	0.022
		PRO	9	15	14	0	0	
		Total	12	36	24	5	0	

Symptom	Week	Group	Yes, Limited a Lot	Yes, Limited a Little	No, not limited at all	*p* -level
Does Health Limit Vigorous Ativities	0	PLA	3	22	14	0.233
		PRO	2	15	21	
		Total	5	37	35	
	6	PLA	3	15	21	0.583
		PRO	2	19	17	
		Total	5	34	38	
	12	PLA	3	19	17	0.558
		PRO	3	14	21	
		Total	6	33	38	
Does Health Limit Moderate Activities	0	PLA	0	3	36	0.665
		PRO	0	4	34	
		Total	0	7	70	
	6	PLA	0	2	37	0.622
		PRO	0	3	35	
		Total	0	5	72	
	12	PLA	0	4	35	0.080
		PRO	1	0	37	
		Total	1	4	72	

Does Health Limit Lifting or Carrying Groceries	0	PLA	1	1	37	0.610
		PRO	0	1	37	
		Total	1	2	74	
	6	PLA	1	0	38	0.320
		PRO	0	0	38	
		Total	1	0	79	
	12	PLA	1	1	37	0.513
		PRO	0	2	36	
		Total	1	3	73	
Does Health Limit Climbing Several Flights of Stairs	0	PLA	0	9	30	0.950
		PRO	0	9	29	
		Total	0	18	59	
	6	PLA	0	6	33	0.358
		PRO	0	9	29	
		Total	0	15	39	
	12	PLA	0	7	32	0.957
		PRO	0	7	31	
		Total	0	14	63	
Does Health Limit Climbing One Flight of Stairs	0	PLA	1	0	38	0.368
		PRO	0	1	37	
		Total	1	1	75	
	6	PLA	1	0	38	0.368
		PRO	0	1	37	
		Total	1	1	75	
	12	PLA	1	2	36	0.219
		PRO	0	0	38	
		Total	1	2	74	
Does Health Limit Bending, Kneeling, or Stooping	0	PLA	1	6	32	0.583
		PRO	0	7	31	
		Total	1	13	63	
	6	PLA	1	4	34	0.564
		PRO	0	3	35	
		Total	1	7	69	
	12	PLA	1	8	30	0.402
		PRO	0	5	33	
		Total	1	13	63	

Does Health Limit Walking More than a Mile	0	PLA	1	5	33	0.301
		PRO	0	2	36	
		Total	1	7	69	
	6	PLA	1	1	37	0.513
		PRO	0	2	36	
		Total	1	3	73	
	12	PLA	1	2	36	0.513
		PRO	0	1	37	
		Total	1	3	73	
Does Health Limit Walking Several Hundred Yards	0	PLA	0	2	37	0.518
		PRO	1	3	34	
		Total	1	5	71	
	6	PLA	0	2	37	0.979
		PRO	0	2	36	
		Total	0	4	73	
	12	PLA	0	0	39	0.147
		PRO	0	2	36	
		Total	0	2	72	
Does Health Limit Walking One Hundred Yards	0	PLA	0	1	37	0.958
		PRO	0	1	38	
		Total	0	2	75	
	6	PLA	0	1	38	0.985
		PRO	0	1	37	
		Total	0	2	75	
	12	PLA	0	1	38	0.320
		PRO	0	0	38	
		Total	0	1	77	
Does Health Limit Bathing or Dressing	0	PLA	1	0	38	0.985
		PRO	1	0	37	
		Total	2	0	77	
	6	PLA	1	0	38	0.368
		PRO	0	1	37	
		Total	1	1	77	
	12	PLA	1	0	38	0.320
		PRO	0	0	38	
		Total	1	0	76	

Symptom	Week		All of the time	Most of the time	Some of the time	A little of the time	None of the time	*p* -level
As a Result of Physical Health / Cut Down on the Amount of Time on Work or Other Activities	0	PLA	1	38	0	0	0	0.594
		PRO	1	36	1	0	0	
		Total	2	74	1	0	0	
	6	PLA	1	38	0	0	0	0.292
		PRO	3	35	0	0	0	
		Total	4	73	0	0	0	
	12	PLA	5	33	1	0	0	0.301
		PRO	2	36	0	0	0	
		Total	7	69	1	0	0	
As a Result of Physical Heatlh / AccomPLAished Less Than Would Like	0	PLA	5	34	0	0	0	0.754
		PRO	4	34	0	0	0	
		Total	9	68	0	0	0	
	6	PLA	6	33	0	0	0	0.961
		PRO	6	32	0	0	0	
		Total	12	65	0	0	0	
	12	PLA	6	33	0	0	0	0.780
		PRO	5	33	0	0	0	
		Total	11	66	0	0	0	
As a Result of Physical Health / Limited in Kind of Work or Other Activities	0	PLA	2	37	0	0	0	0.979
		PRO	2	36	0	0	0	
		Total	4	73	0	0	0	
	6	PLA	2	37	0	0	0	0.622
		PRO	3	35	0	0	0	
		Total	5	72	0	0	0	
	12	PLA	4	35	0	0	0	0.719
		PRO	3	35	0	0	0	
		Total	7	70	0	0	0	
As a Result of Physical Health / Had Difficulty Performing Work or Other Activities	0	PLA	5	34	0	0	0	0.249
		PRO	2	36	0	0	0	
		Total	7	70	0	0	0	
	6	PLA	3	36	0	0	0	0.432
		PRO	5	33	0	0	0	
		Total	8	69	0	0	0	
	12	PLA	1	38	0	0	0	0.156
		PRO	4	34	0	0	0	
		Total	5	72	0	0	0	

As a Result of any Emotional Problems / Cut Down on Time Spent on Work or Other Activities	0	PLA	7	32	0	0	0	0.535
		PRO	9	29	0	0	0	
		Total	16	61	0	0	0	
	6	PLA	3	36	0	0	0	0.432
		PRO	5	33	0	0	0	
		Total	8	69	0	0	0	
	12	PLA	4	35	0	0	0	0.969
		PRO	4	34	0	0	0	
		Total	8	69	0	0	0	
As a Result of any Emotional Problems / AccomPLAished Less Than Would Like	0	PLA	15	24	0	0	0	0.527
		PRO	12	26	0	0	0	
		Total	27	50	0	0	0	
	6	PLA	11	14	0	0	0	0.418
		PRO	14	24	0	0	0	
		Total	39	38	0	0	0	
	12	PLA	6	33	0	0	0	0.093
		PRO	12	26	0	0	0	
		Total	18	59	0	0	0	
As a Result of any Emotional Problems / Did Work or Other Activities Less Carefully Than Usual	0	PLA	11	28	0	0	0	0.104
		PRO	5	33	0	0	0	
		Total	16	61	0	0	0	
	6	PLA	7	32	0	0	0	0.800
		PRO	6	32	0	0	0	
		Total	13	64	0	0	0	
	12	PLA	7	32	0	0	0	0.352
		PRO	4	34	0	0	0	
		Total	11	66	0	0	0	

Symptom	Week		Not at all	Slightly	Moderately	Quite a Bit	Extremely	*p*-level
What Extent Has Physical or Emotional Problems Interferred with Social Activities	0	PLA	18	14	7	0	0	0.012
		PRO	30	5	3	0	0	
		Total	48	19	10	0	0	
	6	PLA	23	10	6	0	0	0.040
		PRO	25	13	0	0	0	
		Total	48	23	6	0	0	
	12	PLA	23	10	5	1	0	0.197
		PRO	22	15	1	0	0	
		Total	45	25	6	1	0	

Symptom	Week		None	Very Mild	Mild	Moderate	Severe	Very Severe	p-level
Bodily Pain During Past Four Weeks	0	PLA	13	15	5	3	3	0	0.600
		PRO	12	18	8	1	1	0	
		Total	25	31	13	4	4	0	
	6	PLA	14	13	9	2	1	0	0.652
		PRO	17	14	5	2	0	0	
		Total	31	27	14	4	1	0	
	12	PLA	14	13	5	6	1	0	0.296
		PRO	17	15	4	1	0	0	
		Total	31	28	9	7	1	0	

Symptom	Week		Not at all	Slightly	Moderately	Quite a Bit	Extremely	*p*-level
How Much Did Pain Interfere with Normal Work	0	PLA	29	7	1	2	0	0.956
		PRO	29	7	1	1	0	
		Total	58	14	2	3	0	
	6	PLA	28	8	3	0	0	0.688
		PRO	30	5	3	0	0	
		Total	58	13	6	0	0	
	12	PLA	26	10	3	0	0	0.646
		PRO	26	7	4	1	0	
		Total	52	17	7	1	0	

Symptom	Week		All of the time	Most of the time	A good bit of the time	Some of the time	A little of the time	None of the time	*p*-level
How Much of the Time do You Feel / Full of Life	0	PLA	1	9	12	13	2	2	0.775
		PRO	1	9	14	11	3	0	
		Total	2	18	26	24	5	2	
	6	PLA	0	10	16	9	1	3	0.070
		PRO	2	14	8	10	4	0	
		Total	2	24	24	19	5	3	
	12	PLA	1	13	14	7	3	1	0.873
		PRO	1	9	18	6	2	2	
		Total	2	22	32	13	5	3	
How Much of the Time do You Feel / Very Nervous	0	PLA	0	3	7	10	16	3	0.374
		PRO	0	5	4	7	14	8	
		Total	0	8	11	17	30	11	
	6	PLA	0	3	4	9	18	5	0.147
		PRO	0	4	3	12	8	11	
		Total	0	7	7	21	26	16	
	12	PLA	1	1	6	11	13	7	0.906
		PRO	1	1	3	9	16	8	
		Total	2	2	9	20	29	15	

How Much of the Time do You Feel / Down in the Dumps	0	PLA	0	1	1	6	9	22	0.783
		PRO	0	0	2	6	11	19	
		Total	0	1	3	12	20	41	
	6	PLA	0	1	2	4	16	16	0.654
		PRO	0	0	1	5	12	20	
		Total	0	1	3	9	28	36	
	12	PLA	0	1	2	4	13	19	0.869
		PRO	0	0	2	4	11	21	
		Total	0	1	4	8	24	40	
How Much of the Time do You Feel / Calm and Peaceful	0	PLA	0	10	13	13	3	0	0.611
		PRO	2	10	13	10	2	1	
		Total	2	20	26	23	5	1	
	6	PLA	0	14	9	14	2	0	0.287
		PRO	2	15	8	8	5	0	
		Total	2	29	17	22	7	0	
	12	PLA	1	15	10	9	3	1	0.891
		PRO	2	15	8	9	4	0	
		Total	3	30	18	18	7	1	
How Much of the Time do You Feel / Have Lots of Energy	0	PLA	0	6	18	10	5	0	0.444
		PRO	1	11	15	6	5	0	
		Total	1	17	28	16	10	0	
	6	PLA	0	9	14	12	3	1	0.350
		PRO	0	14	11	6	6	1	
		Total	0	23	25	18	9	2	
	12	PLA	1	9	18	9	2	0	0.682
		PRO	1	11	16	5	4	1	
		Total	2	20	34	14	6	1	
How Much of the Time do You Feel / Downhearted and Depressed	0	PLA	0	1	2	6	21	9	0.282
		PRO	0	1	3	3	14	17	
		Total	0	2	5	9	35	26	
	6	PLA	0	1	2	8	12	16	0.652
		PRO	0	0	1	5	12	20	
		Total	0	1	3	13	24	36	
	12	PLA	0	2	1	8	15	13	0.346
		PRO	0	0	2	5	12	19	
		Total	0	2	3	13	27	29	

How Much of the Time do You Feel / Worn Out	0	PLA	0	0	7	17	11	2	0.553
		PRO	0	4	7	10	14	3	
		Total	0	6	14	27	25	5	
	6	PLA	0	2	5	17	13	2	0.196
		PRO	0	3	5	7	20	3	
		Total	0	5	10	24	33	5	
	12	PLA	0	2	4	18	13	2	0.396
		PRO	1	1	8	14	9	3	
		Total	1	3	12	32	22	5	
How Much of the Time do You Feel / Happy	0	PLA	0	17	18	2	1	1	0.230
		PRO	1	21	8	5	2	1	
		Total	1	38	26	7	3	2	
	6	PLA	0	19	14	5	1	0	0.215
		PRO	5	18	10	4	1	0	
		Total	5	37	24	9	2	0	
	12	PLA	0	22	12	3	2	0	0.160
		PRO	0	14	15	4	1	0	
		Total	0	36	27	7	3	0	
How Much of the Time do You Feel / Tired	0	PLA	0	10	9	13	7	0	0.561
		PRO	1	5	8	15	8	1	
		Total	1	15	13	28	15	1	
	6	PLA	0	8	9	13	9	0	0.533
		PRO	0	6	8	10	12	2	
		Total	0	14	17	23	21	2	
	12	PLA	1	4	12	11	10	1	0.889
		PRO	3	3	10	11	9	2	
		Total	4	7	22	22	19	3	
How Much of the Time has Emotional or Physical Problems Interferred with Social Activities	0	PLA	0	1	7	8	23	0	0.408
		PRO	0	0	3	10	25	0	
		Total	0	1	10	18	48	0	
	6	PLA	0	1	6	13	18	1	0.138
		PRO	0	1	2	7	28	0	
		Total	0	2	8	20	46	1	
	12	PLA	0	3	6	11	19	0	0.230
		PRO	0	0	3	10	24	1	
		Total	0	3	9	21	43	1	

Symptom	Week		Definitely True	Mostly True	Do not Know	Mostly False	Definitely False	*p* -level
How True or False / I get Sick Easier than Others	0	PLA	2	4	3	18	12	0.313
		PRO	0	4	6	12	16	
		Total	2	8	9	30	28	
	6	PLA	1	5	7	13	13	0.576
		PRO	1	3	3	13	18	
		Total	2	8	10	26	31	
	12	PLA	1	5	7	15	11	0.761
		PRO	1	5	5	11	16	
		Total	2	10	12	26	26	
How True or False / I am as Healthy as Anybody	0	PLA	3	22	8	5	1	0.221
		PRO	10	18	7	3	0	
		Total	13	40	15	8	1	
	6	PLA	4	23	10	2	0	0.227
		PRO	8	21	4	4	1	
		Total	12	44	14	6	1	
	12	PLA	0	22	7	6	1	
		PRO	10	19	5	3	1	
		Total	13	41	12	9	2	
How True or False / I Expect my Health to get Worse	0	PLA	0	7	10	8	14	0.245
		PRO	0	2	11	13	12	
		Total	0	9	21	21	26	
	6	PLA	0	2	7	17	13	0.742
		PRO	0	1	9	13	15	
		Total	0	3	16	30	27	
	12	PLA	0	6	10	16	7	0.318
		PRO	0	2	7	18	11	
		Total	0	8	17	34	18	
How True or False / My Health is Excellent	0	PLA	0	21	6	8	4	0.090
		PRO	4	21	6	7	0	
		Total	4	42	12	15	4	
	6	PLA	1	26	5	7	0	0.254
		PRO	5	21	7	4	1	
		Total	6	47	12	11	1	
	12	PLA	3	27	3	6	0	0.067
		PRO	6	19	10	3	0	
		Total	9	46	13	9	0	

Data are presented as frequencies. PLA = placebo, PRO = plant probiotic. Statistical significance is detailed by chi-squared analysis; $p < 0.05$ considered significant and * denotes significance between groups. † represents statistical tendency ($p > 0.05$ to $p < 0.10$)

Table S14a. Frequency of Side Effects

Symptom	Week	Group	N	None	1-2x's per week	3-4x's per week	5-6x's per week	7-8x's per week	>9x's per week	χ^2 p-level
					Rating of Symptom Frequency					
Gastrointestinal Distress	0	PLA	*39*	26	7	6	0	0	0	0.382
		PRO	*38*	28	7	2	0	1	0	
		Time	*77*	54	14	8	0	0	0	
	6	PLA	*39*	27	10	1	1	0	0	0.543
		PRO	*38*	30	5	2	1	0	0	
		Time	*77*	57	15	3	2	0	0	
	12	PLA	*39*	27	8	4	0	0	0	0.424
		PRO	*38*	28	7	1	1	1	0	
		Time	*77*	55	15	5	1	1	0	
Constipation	0	PLA	*39*	32	4	1	1	1	0	0.648
		PRO	*38*	33	3	2	0	0	0	
		Time	*77*	65	7	3	1	1	0	
	6	PLA	*39*	26	7	3	2	0	1	0.627
		PRO	*38*	24	6	6	1	1	0	
		Time	*77*	50	13	9	3	1	1	
	12	PLA	*39*	28	6	3	0	1	0	0.874
		PRO	*38*	26	7	3	1	1	0	
		Time	*77*	55	13	6	1	2	0	
Diarhhea	0	PLA	*39*	28	9	2	0	0	0	0.726
		PRO	*38*	30	7	1	0	0	0	
		Total	*77*	58	16	3	0	0	0	
	6	PLA	*39*	27	8	2	2	0	0	0.340
		PRO	*38*	26	7	5	0	0	0	
		Total	*77*	53	15	7	2	0	0	
	12	PLA	*39*	27	8	4	0	0	0	0.700
		PRO	*38*	27	9	2	0	0	0	
		Total	*77*	54	17	0	0	0	0	
Fatigue	0	PLA	*39*	16	11	5	2	4	1	0.850
		PRO	*38*	16	8	7	3	4	0	
		Total	*77*	32	19	12	5	8	1	
	6	PLA	*39*	17	13	7	1	1	0	0.692
		PRO	*38*	12	16	6	1	3	0	
		Total	*77*	29	29	13	2	4	0	
	12	PLA	*39*	15	13	6	3	2	0	
		PRO	*38*	15	13	3	3	3	1	
		Total	*77*	30	26	9	6	5	1	
Abdominal Discomfort	0	PLA	*39*	32	5	1	1	0	0	0.376
		PRO	*38*	27	8	3	0	0	0	
		Total	*77*	59	13	4	1	0	0	
	6	PLA	*39*	29	7	2	1	0	0	0.989
		PRO	*38*	27	8	2	1	0	0	
		Total	*77*	56	15	4	2	0	0	
	12	PLA	*39*	32	5	0	1	1	0	0.370
		PRO	*38*	26	10	1	1	0	0	
		Total	*77*	58	15	1	2	1	0	

Nausea	0	PLA	*39*	35	2	1	1	0	0	0.721
		PRO	*38*	36	1	1	0	0	0	
		Total	*77*	71	3	2	1	0	0	
	6	PLA	*39*	33	6	0	0	0	0	0.135
		PRO	*38*	34	2	2	0	0	0	
		Total	*77*	67	8	2	0	0	0	
	12	PLA	*39*	32	5	0	1	1	0	0.471
		PRO	*38*	34	3	1	0	0	0	
		Total	*77*	66	8	1	1	1	0	
Headache	0	PLA	*39*	20	13	2	3	1	0	0.417
		PRO	*38*	22	11	4	0	1	0	
		Total	*77*	42	24	6	3	2	0	
	6	PLA	*39*	17	15	3	4	0	0	0.174
		PRO	*38*	24	9	1	2	2	0	
		Total	*77*	41	24	4	6	2	0	
	12	PLA	*39*	22	10	5	1	1	0	0.854
		PRO	*38*	21	10	5	2	0	0	
		Total	*77*	43	20	10	3	1	0	
Heartburn	0	PLA	*39*	34	3	2	0	0	0	0.629
		PRO	*38*	30	5	2	0	1	0	
		Total	*77*	64	8	4	0	1	0	
	6	PLA	*39*	31	4	3	1	0	0	0.911
		PRO	*38*	30	4	2	2	0	0	
		Total	*77*	61	8	5	3	0	0	
	12	PLA	*39*	31	4	2	1	1	0	0.711
		PRO	*38*	32	1	2	2	1	0	
		Total	*77*	63	5	4	3	2	0	

Data are presented as frequencies for the placebo (PLA) and plant probiotic (PRO) groups. Statistical significance is detailed by chi-squared analysis; $p \leq 0.05$ considered significant while $p > 0.05$ to $p < 0.10$ are considered approaching significance. Totals are included but, separate from analysis.

Table S14b. Severity of Side Effects

Symptom	Week	Group	N	None	Minimal	Slight	Moderate	Severe	Very Severe	χ^2 p-level
				Rating of Symptom Severity						
Gastrointestinal Distress	0	PLA	*39*	27	3	7	2	0	0	0.244
		PRO	*38*	29	6	2	1	0	0	
		Time	*77*	56	9	9	0	0	0	
	6	PLA	*39*	28	10	0	1	0	0	0.197
		PRO	*38*	29	5	3	1	0	0	
		Time	*77*	57	15	3	2	0	0	
	12	PLA	*39*	30	6	2	1	0	0	0.937
		PRO	*38*	29	7	1	1	0	0	
		Time	*77*	59	13	3	2	0	0	
Constipation	0	PLA	*39*	32	3	1	3	0	0	0.240
		PRO	*38*	33	2	3	0	0	0	
		Time	*77*	65	5	4	3	0	0	
	6	PLA	*39*	26	9	3	1	0	0	0.771
		PRO	*38*	24	8	3	3	0	0	
		Time	*77*	50	17	6	4	0	0	
	12	PLA	*39*	28	9	1	1	0	0	0.585
		PRO	*38*	27	6	3	2	0	0	
		Time	*77*	55	15	4	3	0	0	
Diarhhea	0	PLA	*39*	29	7	3	0	0	0	0.605
		PRO	*38*	30	7	1	0	0	0	
		Total	*77*	59	14	4	0	0	0	
	6	PLA	*39*	27	9	1	2	0	0	0.772
		PRO	*38*	25	8	3	2	0	0	
		Total	*77*	52	17	4	4	0	0	
	12	PLA	*39*	28	8	1	1	1	0	0.663
		PRO	*38*	27	9	2	0	0	0	
		Total	*77*	55	17	3	1	1	0	
Fatigue	0	PLA	*39*	16	10	5	5	3	0	0.263
		PRO	*38*	18	6	9	5	0	0	
		Total	*77*	34	16	14	10	3	0	
	6	PLA	*39*	17	12	6	3	1	0	0.393
		PRO	*38*	19	6	10	3	0	0	
		Total	*77*	36	18	16	6	1	0	
	12	PLA	*39*	16	7	8	8	0	0	0.422
		PRO	*38*	15	10	9	3	1	0	
		Total	*77*	31	17	17	11	1	0	
Abdominal Discomfort	0	PLA	*39*	32	4	2	1	0	0	0.719
		PRO	*38*	27	6	3	2	0	0	
		Total	*77*	59	10	5	3	0	0	
	6	PLA	*39*	28	8	3	0	0	0	0.518
		PRO	*38*	27	7	2	2	0	0	
		Total	*77*	55	15	5	2	0	0	
	12	PLA	*39*	33	2	2	2	0	0	0.094
		PRO	*38*	27	8	3	0	0	0	
		Total	*77*	60	10	5	2	0	0	

Nausea	0	PLA	*39*	35	2	0	1	1	0	0.572
		PRO	*38*	36	2	0	0	0	0	
		Total	*77*	71	4	0	1	0	0	
	6	PLA	*39*	33	4	2	0	0	0	0.247
		PRO	*38*	34	1	1	2	0	0	
		Total	*77*	67	5	3	2	0	0	
	12	PLA	*39*	32	2	3	0	1	1	0.256
		PRO	*38*	35	3	0	0	0	0	
		Total	*77*	67	5	3	0	1	1	
Headache	0	PLA	*39*	19	10	4	5	1	0	0.716
		PRO	*38*	22	6	6	6	1	0	
		Total	*77*	41	16	10	8	2	0	
	6	PLA	*39*	17	13	7	2	0	0	0.054
		PRO	*38*	26	4	5	1	2	0	
		Total	*77*	43	17	12	3	2	0	
	12	PLA	*39*	21	5	5	4	0	1	0.550
		PRO	*38*	21	9	5	2	1	0	
		Total	*77*	45	14	10	6	1	1	
Heartburn	0	PLA	*39*	33	4	1	1	0	0	0.658
		PRO	*38*	30	3	2	3	0	0	
		Total	*77*	63	7	3	4	0	0	
	6	PLA	*39*	31	5	3	0	0	0	0.377
		PRO	*38*	31	4	1	2	0	0	
		Total	*77*	62	9	4	2	0	0	
	12	PLA	*39*	30	4	1	3	1	0	0.845
		PRO	*38*	32	3	1	2	0	0	
		Total	*77*	62	7	2	5	1	0	

Data are presented as frequencies for the placebo (PLA) and plant probiotic (PRO) groups. Statistical significance is detailed by chi-squared analysis; $p \leq 0.05$ considered significant while $p > 0.05$ to $p < 0.10$ are considered approaching significance. Totals are included but, separate from analysis.
